# Quercetin instability in red wines: Insights into precipitation and control approaches

**DOI:** 10.1016/j.crfs.2026.101319

**Published:** 2026-01-22

**Authors:** Alessandra Luciano, Luigi Moio, Angelita Gambuti

**Affiliations:** Department of Agricultural Sciences, Section of Vine and Wine Sciences, University of Napoli ″Federico II″, Viale Italia, Avellino, 83100, Italy

## Abstract

One effect of climate change on grape composition is the increased biosynthesis of quercetin and its glycosides in response to UV-B radiation. High concentrations of quercetin in wine can exceed its solubility threshold, resulting in undesirable precipitation that negatively impacts clarity and consumer perception. This poses a challenge to producers in terms of both quality and economics. Starting from a survey of quercetin instability and its presence in wines, this review summarises the current understanding of the biochemical and physicochemical mechanisms that regulate quercetin stability and precipitation in red wines. It also covers the factors involved in quercetin extraction and stability during vinification and oenological strategies to counteract these effects.

Taken together, these findings demonstrate that the stability of quercetin in red wines is governed by a complex interplay of factors, including anthocyanin-mediated copigmentation, pH, temperature, nucleation seeds and the broader phenolic composition of the wine matrix. By elucidating these mechanisms, the review provides a framework for predicting and managing quercetin precipitation, thereby supporting improved wine quality and colloidal stability in the challenging conditions imposed by climate change.

## Highlights

• Excess quercetin in wine may cause haze and appearance of deposits.
• Solubility of quercetin in red wines depends on matrix composition.
• Sangiovese is the grape variety most sensitive to the risk of quercetin deposits.
• Micro-oxygenation, nucleation seeds, PVPP and charcoal decrease quercetin in wines.

## Graphical abstract

**Figure:**
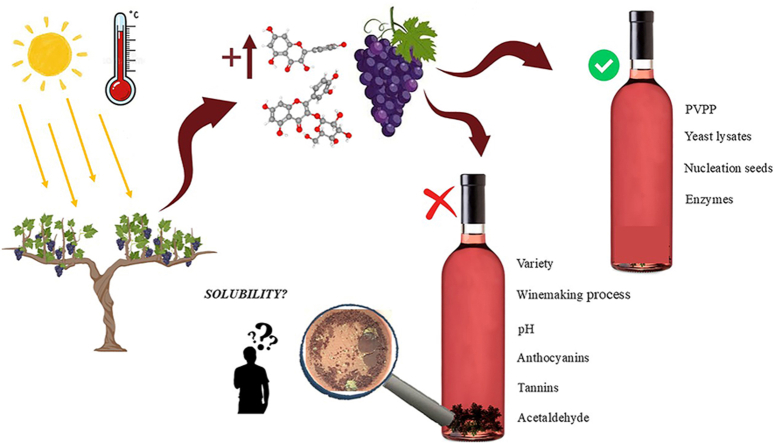

## 1 Introduction

In recent years, anomalous precipitation phenomena have been observed in red wines, often attributed to excessive quercetin concentrations (Lanati et al., 2021). This molecule belongs to the flavonol class and is synthesised via the phenylpropanoid pathway. Although flavonols such as kaempferol, quercetin, myricetin and isorhamnetin are present in finished wines, they primarily exist in grape berries as their respective glucosides, glucuronides, galactosides and rutinosides.

In *Vitis vinifera* cultivars, including Pinot Noir, Shiraz, Merlot, Sangiovese and Nebbiolo, the concentration of these compounds depends heavily on the light exposure of the tissues in which they accumulate (Price et al., 1995; Spayd et al., 2002; Downey et al., 2004; Lanati et al., 2021; Wilson et al., 2024). More specific studies on genetic expression have reported that the biosynthesis of quercetin is strictly controlled at the transcriptional level and is highly sensitive to external climatic factors, particularly light intensity and temperature extremes (as reviewed by Ferreyra et al., 2021; Cao et al., 2024; Laoué et al., 2022). Furthermore, elevated levels of environmental stressors, such as increased salinity and calcium content, act as inductors that enhance synthesis (Shomali et al., 2022; Martins et al., 2021).

Unfortunately, exposure to UV-B radiation, temperature extremes and increased salinity are direct consequences of climate change (Van Leeuwen and Darriet, 2016). These stressors often lead to increased accumulation of flavonol glycosides in sensitive grapevine cultivars (Gambuti et al., 2020). Subsequent transfer of these hyper-accumulated molecules into the wine matrix, followed by hydrolysis over time, results in concentrations of the aglycone (specifically quercetin) that exceed its solubility limit. Ultimately, this supersaturation leads to the precipitation of excess quercetin, which can occur even after bottling and commercialisation.

Although it is now clear that this mechanism specifically, a high initial concentration of glycosides followed by delayed hydrolysis causes the unusual precipitates observed in bottled wines, particularly varieties such as Sangiovese, several aspects remain unclear. Consequently, developing systematic and reliable oenological strategies to mitigate this issue remains a significant challenge. This difficulty is linked to the following key limitations. Firstly, the solubility of quercetin varies significantly within different wine matrices, so this must be considered. Secondly, risk assessment cannot rely solely on aglycone content. In fact, a comprehensive predictive model should integrate initial flavonol glycoside levels and their subsequent hydrolysis rates at different wine pH levels. Furthermore, existing studies and comprehensive reviews evaluating the transfer and evolution of phenolic compounds from grapes to wine have historically focused on major components such as anthocyanins and tannins, often neglecting the flavonol class of compounds.

Therefore, the present state of the art aims to address this knowledge gap. Using the current understanding of quercetin solubility as a starting point, this work will systematically analyse all relevant technological and oenological factors, from grape cultivar and harvest timing to wine ageing protocols. Furthermore, the review will outline current knowledge regarding the efficacy of traditional practices (e.g. the use of fining agents and additives) and innovative techniques (e.g. the application of imprinted polymers) in managing quercetin and its glycoside content.

## 2 Brief overview of the roles of quercetin

Quercetin is a widely distributed bioactive molecule found not only in grapes and wines but in all the plant kingdom. Its diverse pharmacological activities, including antioxidant, antiviral, immunomodulatory and anticancer properties, have made it the subject of extensive research (Aghababei et al., 2023). To our knowledge, only in 2025, 12 extensive reviews have been published on quercetin, highlighting its potential across diverse biological and industrial applications, primarily driven by its potent antioxidant and antimicrobial properties (source: Scopus database). Current research focuses on the impact of quercetin in areas ranging from food preservation to neurodegenerative diseases (Zhao et al., 2025). Jalouli et al. (2025), for example, reviewed the potential of quercetin in mitigating the progression of neurodegenerative disorders such as Alzheimer disease, Parkinson disease, and multiple sclerosis. Meanwhile, Esmaeli et al. (2025) mainly investigated its anticancer effects in relation to the dysregulated PI3K-AKT-mTOR signalling pathway, which plays a crucial role in tumour growth and survival in cancers such as breast, prostate and brain. Furthermore, quercetin has been shown to have a protective effect against hyperuricemia (Bian et al., 2025) and to mitigate arthritis (Chaudhary et al., 2025). Additionally, quercetin-polysaccharide-based hydrogels promote tissue repair by gradually releasing quercetin, which stimulates collagen deposition and angiogenesis, alleviates chronic inflammation and reduces oxidative stress at the wound site (Ashfaq et al., 2025).

Beyond their recognized health properties, quercetin and its glycosides play a critical role in wine chemistry as co-pigments that enhance the colour expression of anthocyanins. Asen et al. (1972) demonstrated that quercetin glycosides (rhamnose and glucose) induce spectral shifts of 15–20 nm, increasing the absorbance of aqueous cyanidin 3,5-diglucoside solutions by 150–200 %. This effect is attributed to their extended π-conjugation, which promotes π–π interactions and stabilises copigmentation interactions (Boulton, 2001). Copigmentation between anthocyanins and flavonols during vinification also facilitates greater anthocyanin extraction (Rustioni et al., 2012).

Additionally, quercetin and its derivatives influence wine sensory attributes, as recently reviewed by Paissoni et al. (2023). Preys et al. (2006) suggested a link between flavonol aglycones (e.g. myricetin and quercetin) and bitterness. Also, flavonol glycosides have been shown to impart a velvety astringency (Hufnagel and Hofmann, 2008) and quercetin-3-*O*-galactoside (0–10 mg/L) has been used as a reference standard for velvety astringency in sensory panel training (Gonzalo-Diago et al., 2014). However, the demarcation between sensations is not so specific or clear: Ferrer-Gallego et al. (2016) reported that quercetin 3-*O*-glucoside increased astringency, roughness, dryness, bitterness and persistence in red wines while imparting greenness and bitterness to white wines. Flavonols in red wine fractions have also been associated with burning and hot sensations (Sáenz-Navajas et al., 2017) and have been identified as a major contributor to grape-derived bitterness (Ferrero-del-Teso et al., 2022). More specifically, regarding their role in the perception of wine astringency, flavonols exhibit the lowest astringency recognition thresholds among polyphenols and, their glycosides lack affinity for salivary proteins, suggesting the existence of alternative perception mechanisms (Scharbert et al., 2004). Guerreiro et al. (2022) demonstrated interactions between flavonol extracts from yellow onions and oral cell lines, particularly those from the tongue. However, it is important to note that, as flavonols are extracted alongside tannins, which dominate the perception of bitterness and astringency, they play a less important role in the overall mouthfeel sensation. Given the precipitation risk of quercetin and its glycosides in wines, as well as their contribution to colour, sensory properties and health benefits, it is necessary to manage these flavonols rather than remove them completely during winemaking.

## 3 Instability of quercetin in red wines

Flavonol instability is, until now related to few varieties and, likely for this reason, studies on this phenomenon are limited as shown in Table 1. To our knowledge the earliest references to the formation of flavonol deposits in wines in the literature dates to 1969; in a winery in Southern Australia the formation of needle-like deposits in a red table wine made from the Shiraz variety (*Vitis vinfera*) was observed. This deposit was insoluble in water but soluble in ethanol, methanol, acetone, and sodium hydroxide. Thin-layer chromatography showed that the deposit was formed by quercetin and kampferol (Ziemelis and Pickering, 1969). The cause of that instability was not determined at the time, and it turned out to be an isolated incident, as no similar instability in red wines was previously observed. In Australia, quercetin and, to a lesser extent, kaempferol deposits were first observed in white wines in 1985 (Somers and Ziemelis, 1985). It was underlined that these deposits can also develop in finished and bottled wines, resulting in a deterioration in product quality. For the first time it was hypothesized that glycosylated flavonols from grapes released quercetin during the first phase of vinification process due to the acidic or enzymatic hydrolysis. On this basis, in that work the nature of this problem by using an enzymatic treatment to assess whether a white wine risked deposit formation was evaluated. If after the treatment the formation of deposits was observed the wine was considered unstable. Then, the unstable wine was filtered or centrifuged, and the sediment was resuspended in methanol. Spectrophotometric analysis showed that the deposit was made of quercetin aglicone (Somers and Ziemelis, 1985). The authors showed that aglycon of quercetin is responsible for the formation of deposits and indicated 5 mg/L as solubility threshold for this molecule in white wine.

**Table 1:** Kind of instability attributed to quercetin, methods of detection and solubility threshold reported in literature for quercetin in model solutions and wine.

Appearance of instability	Grape variety	Medium	Methods of detection	Solubility Threshold	References
Deposits of fine-needle shaped crystals	Shiraz	Dry red table wine	X-ray diffraction and Thin-layer chromatography		Ziemelis and Pickering (1969)
Yellow deposit of flavonols being principally quercetin with some kampferol	/	White wine	Microscopic examination	5 mg/L	Ziemelis (1982).
Yellow deposits in white juice and wines	/	White wine	Spectrophotometry and HPLC	3–4 mg/L in wine	Somers and Ziemelis (1985)
Voluminous precipitate of quercetin aglycon	/	Red wine	HPLC–DAD Analysis of Flavonols, spectrophotometric analysis (recording the absorption spectrum from 230 to 700 nm)		Lanati et al., (2014)
Wine cloudy over time due to the formation of a precipitate	Sangiovese, Aglianico, Cabernet Sauvignon, Nerello Mascalese, Primitivo, Nero di Troia, Pallagrello, Casavecchia,Magliocco, Syrah,Merlot, Nero d'Avola	Red Wine/pH 3.20 buffer solution	Microscopic analysis, HPLC–DAD Analysis of Flavonols, NMR analysis	0.4 μg/mL in water at pH 3; *(Li et al. 2013)*3mg/L *in* hydro-alcoholic solutions and red wines 3 mgL/L	Gambuti et al., (2020)
Quercetin haze	Barbera 2018, red Cirò 2014, Sangiovese 2014	Red Wine/pH 3.20 buffer solution	HPLC-DAD Analysis of supernatant	1.64 mg/L at 18 °C and 1.16 mg/L at 0 °C in hydroalcoholic solution, 27.8 mg/L at 18 °C in Barbera wine, 18.9 mg/L at 0 °C in the same Barbera wine, 16.0 mg/L at 18 °C in red Cirò and 12.6 mg/L at 0 °C in the same red Cirò, 8.8 mg/L at 18 °C in Sangiovese wine and 7.7 mg/L at 0 °C in the same Sagiovese wine, 7.30 mg/L at 18 °C in white Cirò and 5.51 mg/L at 0°Cin the same white Cirò	Lanati et al., (2022)
Elaboration of a precipitation risk index	Sangiovese from different vintages 2017, 2018 and 2019	Red Wine	HPLC UV–VIS Analysis and HRMS mass spectrometer	15 mg/L in stable wine *(Biondi Bartolini, 2018)*	Borghini (2022)
Sediment	Sangiovese	Red Wine/pH 3.20 buffer solution	Spectrophotometry and HPLC of supernatant	3 mg/L in red wine	Luciano et al., (2024)
Sediment at the bottom of the bottle	Aglianico, Barbera, Camaiola,Casavecchia, Dolcetto,Merlot, Montepulciano, Nebbiolo,Negroamaro, Nero d’Avola, Nero di Troia, Pallagrello Nero, Piedirosso, Primitivo,Sangiovese, Susumaniello,Teroldego.	Red wines	Spectrophotometry and HPLC of supernatant		Luciano et al., (2025)

Authors attributed the high quercetin content and the resulting formation of deposits to various winemaking practices, such as the presence of a significant amount of leaves in the must due to mechanical harvesting, the heavy pressing of grapes, and the use of high doses of SO_2_, which facilitate the extraction of glycosides and subsequent potential deposit formation. However, the removal of leaves during winemaking fixed the instability issue. Therefore, the main cause of flavonol instability was attributed to the excessive presence of leaves, which was caused by the widespread adoption of mechanical harvesting. Indeed, Somers (1985) have already demonstrated that vine leaf extracts contain high concentrations of quercetin glycosides, primarily in the form of 3-*O*-glucuronides, rutinosides, and glucosides.

Other studies on the formation of quercetin deposits in red wines only emerged in 2014 (Lanati et al., 2014), likely because modern harvesting techniques reduced leaf contamination in musts, thereby limiting the presence of flavonol glycosides in wines. It is also possible that, in recent years, due to climate change, the issue of phenolic instability and the formation of quercetin deposits in red wines has become increasingly significant due to the strong impact of solar radiation on flavonols biosynthesis in the berry (Price et al., 1995). In 2014, Lanati et al., firstly underlined that the voluminous precipitates of quercetin in wines, caused economic losses for wine producers.

Since 2020, numerous studies have explored the quercetin issue in Sangiovese and other grape varieties, focusing on the influence of different phenolic compositions of wines and potential resolution strategies. Gambuti et al. (2020) explores the influence of vintage on quercetin content in wines, confirming that environmental factors, particularly solar radiation, and temperature, may play a significant role. The study found that among the various vintages, wines from the 2017 season, which experienced the highest levels of solar radiation and elevated temperatures, had the highest quercetin content. Additionally, the aging process was identified as a contributing factor to the levels of these compounds. Although Sangiovese is identified as the variety richest in quercetin and quercetin glycosides, other minor varieties also show high levels of quercetin and quercetin glycosides, yet quercetin precipitation is not observed in these varieties (Gambuti et al., 2020; Simonetti et al., 2022) This discrepancy has led to new hypotheses and further investigations on factors affecting quercetin solubility in red wine as there is a wide range of reported solubility values for quercetin in wine. In a previous study the possibility of defining a solubility range was evaluated by spiking eleven hydroalcoholic solutions and eleven real wines with increasing concentrations of quercetin (Gambuti et al., 2020). This experiment showed that there is a correlation between the solubility of quercetin and the matrix, because in the hydroalcoholic solution there was the formation of precipitate after 10 days, while in the real wine it takes more time to form the deposits.

### 3.1 Chemical nature of flavonol deposits

**Fig. 1:**
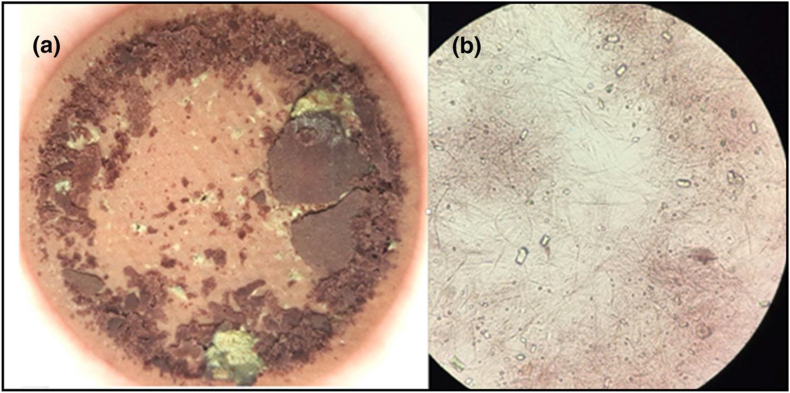
Characteristics of the deposit obtained from Sangiovese wines. a visual aspect of the deposit on the filter, b microscopic analysis of the precipitate (Figure reproduced with permission from Gambuti et al., 2020).

**Fig. 2:**
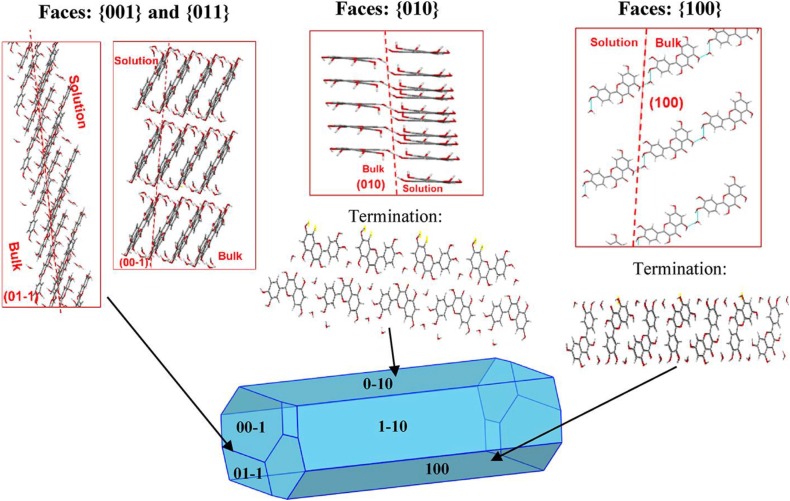
Surface chemistry analysis schematic for quercetin dihydrate showing the growth intermolecular interactions by which the {010}, {100}, {001, and {011} habit planes of quercetin dihydrate grow. Light blue lines indicate hydrogen bonds. Reprinted from Klitou et al. (2022) CC-BY 4.0 (https://creativecommons.org/licenses/by/4.0/).

The microscopic image of precipitates in unstable wines, revealed needle-shaped crystals (Gambuti et al., 2020) see Fig. 1. To confirm that these crystals were made of quercetin, a qualitative analysis was performed using ^1^H NMR and ^13^C-NMR on deposits filtered from cloudy Sangiovese wines. The analysis demonstrated that the deposits consisted of quercetin crystals, with kaempferol detected in only one sample (Gambuti et al., 2020). Based on literature, the aglycon gives rise to crystals due to its planar conformation in which the molecules tend to be closely stacked due to strong π-π interactions (Klitou et al., 2022). The crystal structure of quercetin has been elucidated by Rossi et al., (1986) and Jin et al., (1990). Quercetin crystallizes in the triclinic space group P1, with two independent molecules present in the asymmetric unit. The morphology of the crystal is influenced by extensive intra- and intermolecular hydrogen bonding, which arises from the presence of multiple oxygen-containing functional groups. These hydrogen-bonding interactions play a critical role in stabilizing the crystal lattice (Rossi et al., 1986; Jin et al., 1990). More detailed description of quercetin crystals was recently provided by Klitou et al. (2022). Their study, which combined modelling results with experimental observations, revealed a facet-specific anisotropy in the surface properties of the crystals (Fig. 2). The researchers identified variations in hydrophobicity, morphology, and polarity among different facets, as well as heterogeneous surface energy across these facets. In their findings on quercetin dihydrate, they noted that some facets are likely hydrophobic due to their growth primarily through nonpolar quercetin–quercetin stacking interactions. The authors also noticed that other facets are also expected to be hydrophilic, as their growth is driven mainly by polar quercetin–water hydrogen bonding. Therefore, the molecular forces beyond the crystal formation are of different nature.

### 3.2 On the solubility of quercetin

One of the fields in which numerous studies of solubility has been carried out is the pharmaceutical. Quercetin being a hydrophobic molecule has relatively low solubility in water (0.17–7 μg/mL), gastric fluids (5.5 μg/mL), and small intestine fluids (28.9 μg/mL) (Kandemir et al., 2022; Kasikci & Bagdatlioglu, 2016). Consequently, this low solubility leads to a tendency for quercetin to precipitate in these fluids, which diminishes its bio-accessibility and, therefore, the amount available for absorption. In wine we have firstly to consider that pH and ethanol affect quercetin solubility. Previous investigations by Abraham et al. (2014) demonstrated that the solubility of quercetin increases proportionally with the ethanol concentration in aqueous solutions. Interestingly, the solubility of quercetin in wine closely mirrors that observed in water, suggesting that the ethanol content is too low to govern quercetin solubility in such matrices. In a more recent study, Gambuti et al. (2020) conducted an in-depth assessment of the solubility and precipitation thresholds of quercetin in both hydroalcoholic solutions and red wines. The authors identified 3 mg/L as a critical concentration above which the risk of quercetin deposit formation becomes significant. Notably, they also highlighted the temporal dependence of quercetin precipitation, indicating that deposit formation increases over time. In agreement with findings of Chebil et al. (2007), the medium in which quercetin is dissolved plays a crucial role in modulating its precipitation. Unlike simple hydroalcoholic solutions, wine represents a complex colloidal system in which several components may affect the nucleation and growth of quercetin crystals. Moreover, non-covalent interactions could contribute to the stabilization of quercetin in solution increasing its solubility. Lanati et al. (2022) evaluated the solubility of quercetin in hydroalcoholic buffer solution, in three different red wines, and in a white wine, by spiking them with 30 mg/L of quercetin and considering different storage temperatures. They observed that solubility was lower at 0 °C compared to 18 °C in all cases. Additionally, results suggested that an observed higher solubility of quercetin in wines can be due to the presence of anthocyanins, which may help to maintain quercetin in solution. The formation of soluble co-pigmentation complexes between quercetin and anthocyanins could prevent quercetin precipitation. These significant findings were confirmed by a recent study (Luciano et al., 2024), which evaluated the effect of increasing anthocyanin concentrations on quercetin solubility in a model solution. The results showed that higher concentrations of anthocyanins in solution corresponded to higher concentrations of quercetin in solution. Another important finding was that this effect decreases over time. A significant contribution to this idea was made by Borghini (2022), who screened over 50 Sangiovese samples from various vintages. The study identified significant positive correlations between quercetin aglycone and anthocyanins, as well as between the bound forms of quercetin and tannins. These findings were partially confirmed by Luciano et al., (2025), who carried out a correlation test on 32 red wines from the 2023 vintage and found a moderate correlation between the amount of soluble quercetin after 8 months of bottle storage and flavonoids detected as vanillin reactive flavans, iron-reactive phenols, bovine serum albumine-reactive tannins and the ratios tannins/monomeric anthocyanins and flavans/monomeric anthocyanins supporting the hypothesis that the presence of these macromolecules may facilitate the solubilization of quercetin and inhibit crystal growth. (Luciano et al., 2025).

## 4 Survey of free and glyco-conjugate quercetin in red wines

**Fig. 3:**
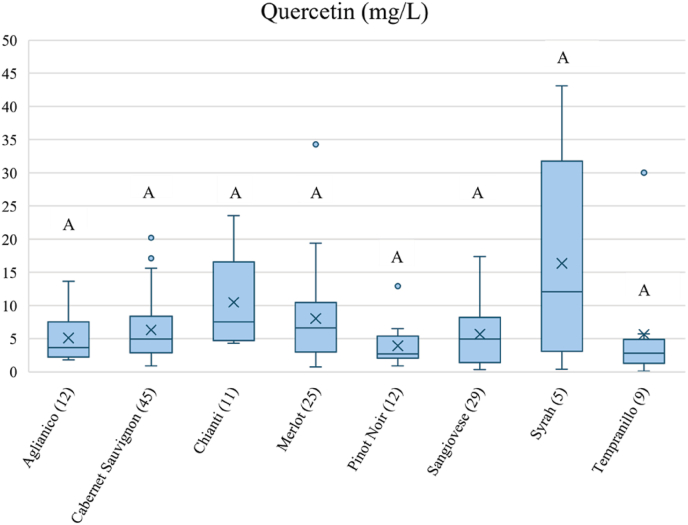
Box plots of quercetin content in wines obtained from different variety/denominations resuming data in literature (Table S1). Medians are shown as central horizontal bars, means as (x), and the box limits indicate the first and third quartiles; points outside the whiskers represent outliers. Box widths have no statistical meaning. The normality of the data was tested using the Shapiro–Wilk test. Due to the non-normal distribution, differences were assessed using the Kruskal–Wallis test was conducted at a 95 % confidence interval with a significance level of 5 % (*p* < 0.05).

**Fig. 4:**
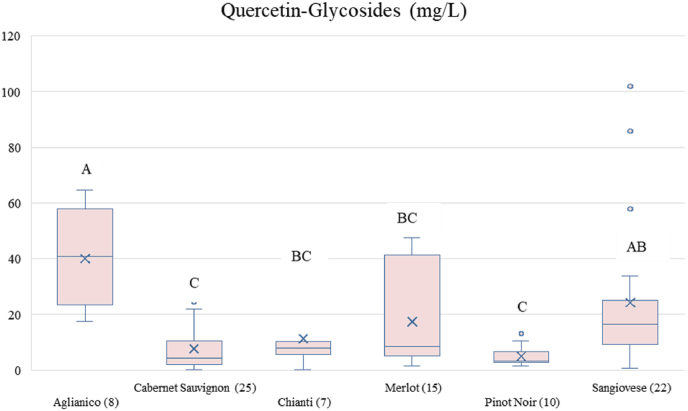
Box plots of quercetin-glycosides content in wines obtained from different variety/denomination resuming data in literature (Table S1). Medians are shown as central horizontal bars, means as (x), and the box limits indicate the first and third quartiles; points outside the whiskers represent outliers. Data normality was tested with Shapiro–Wilk, and, due to normal distribution, differences were assessed using the Tukey-test. Different letters mean significant differences at p < 0.05.

The content of quercetin and its glycosides in wine are strongly influenced by grape variety, geographical origin, climate conditions, and winemaking practices, leading to significant variations in their levels across different wines (Fig. 3, Fig. 4 and Table S1).

One of the first data on the quantification of quercetin in red wines dates to 1984 (Salagoïty-Auguste and Bertrand, 1984), when Salagoity evaluated the concentration of low molecular weight phenolic compounds in three Bordeaux wines from 1982, after extraction with ethyl acetate at different pH levels (Fig. 2). The quercetin values of extracts obtained from the two red wines analysed were for Cabernet Sauvignon 10.4 mg/L and for the Merlot 14.8 mg/L. Of the different varieties analysed and documented in the literature, the Cabernet Sauvignon is one of the most widely studied, given that it is the most widely cultivated international variety. A great variability can be observed among Cabernet Sauvignon wines originating from different regions and vintages. We observed a minimum quercetin concentration of 0.9 mg/L in a Cabernet from Chile (McDonald et al., 1998) and a maximum of 15,6 mg/L (excluding outliers) in a Cabernet from California (Frankel et al., 1995), with an overall mean value of 4.93 mg/L on a total of 45 samples analysed. Among the Merlot wines (25 samples), the mean quercetin concentration is 6,6 mg/L (excluding outliers). The maximum observed value is 34.3 mg/L (Vinas et al., 2000), which is notably high compared to all other varieties. Thus, Merlot generally shows higher quercetin levels compared to Cabernet. In the same article of Viñas (2000) a high quercetin concentration was observed also for Syrah (43.1 mg/L) compared to other Syrah wines reported in literature (Table S1). However, in that study, Viñas (Vinas et al., 2000) analysed different phenols in wines using liquid chromatography with photodiode array and fluorescence detection. Regarding Syrah, across 5 samples the mean quercetin concentration is 12.07 mg/L, with a maximum value of 43.1 mg/L detected in a Syrah wine from the 1997 vintage. It is interesting to observe that in the work of Viñas (2000), a group of commercially available wines was analysed, including one Tempranillo from the 1997 vintage, which showed high levels of quercetin when compared with literature (Table S1). Regarding the available data on the Tempranillo variety, we have limited information on quercetin content with a mean value of 2.8 mg/L on a total of nine wines analysed (excluding outliers). The fact that these wines were produced in 1997 and have higher quercetin content with respect to wines produced from the same grape varieties in other years, highlights that vintage can significantly impact quercetin levels. This also underscores the importance of considering the year of production when studying the phenolic profiles of wines, as certain conditions may favour the accumulation of quercetin and other phenolic compounds. However, it is also fundamental to consider the aging time of wines before the analysis was made, and the method of analysis used. Other data are related to Pinot Noir: the values of quercetin in these wines vary considerably, ranging from 12.9 mg/L for a Californian Pinot from the 1992 vintage to 0.9 mg/L for a French Pinot Noir from 1983 (McDonald et al., 1998). Regarding the quercetin content in Sangiovese, a variety that in recent years has been at the centre of issues related to the formation of quercetin deposits in the bottle, the values are not different from other varieties (0.3 mg/L-17 mg/L) when data on monovarietal wines are analysed. However, data on Chianti and Chianti Classico, two prestigious Tuscan denominations that mandate a minimum percentage of Sangiovese (70 % for Chianti and 80 % for Chianti Classico) show different results: the mean quercetin values in Chianti samples are higher than those of other previously evaluated cultivars, with even the minimum values exceeding those of other wines (see Table S1). This suggests that these high-quality wines were produced using viticultural practices that focused on enriching the grapes with phenolic compounds, resulting in a higher accumulation of quercetin. Alternatively, the higher concentration of quercetin could be due to the contribution of other grapes authorised for Chianti production, even if their contribution is lower than 20 %. However, it is worth noting that a smaller number of samples were analysed in this instance.

Considering quercetin conjugates, as summarized in Fig. 4 and Table S1, their concentrations vary considerably reflecting the influence of both genetical and environmental factors on flavonol biosynthesis. Beyond their analytical significance, quercetin glycosides have important implications for wine chemistry and bioactivity. Glycosylation enhances water solubility and modulates the stability of quercetin, while enzymatic hydrolysis during fermentation and aging can liberate the aglycone, altering the phenolic profile of the wine. These transformations may affect the chemical properties and ageing potential of wines. It is important to note that literature consistently reports the presence of rutin, quercetin-3-*O*-glucuronide, quercetin-3-*O*-glucoside, and in some cases, quercetin rhamnoside (Table S1). Quercetin-3-*O*-galactoside has not been identified in these studies, despite its detection in more recent works. It is also crucial to mention that in many studies, the glycosidic fraction is evaluated following acid hydrolysis and is often considered collectively as a mixture of glycosides, without distinguishing the individual glycosides present. This approach can hide the specific profile of quercetin glycosides in the wines, underscoring the need for more detailed analyses to identify each glycoside distinctly. In Montepulciano d’Abruzzo wines, multiple quercetin glycosides have been detected, including quercitrin (0.20 mg/L)**,** quercetin-3-*O*-glucoside (0.63 mg/L), and quercetin-3-*O*-rutinoside (0.03 mg/L) (Simonetti et al., 2022)**,** illustrating a diverse glycosidic profile even within a single cultivar. In contrast, Barbera wines generally exhibit lower concentrations, with quercitrin typically around 0.02 mg/L and quercetin-3-*O*-glucuronide near 0.6 mg/L, although quercetin-3-*O*-glucoside can still reach 1.81 mg/L in certain vintages. Significantly higher concentrations are reported in Agiorgitiko wines from the Peloponnese, where quercetin-3-*O*-rutinoside ranges from 8.5 to 44.2 mg/L and quercetin-3-*O*-rhamnoside reaches 26.0–96.2 mg/L, (Arnous et al., 2001). suggesting that grape genotype as well as geographical origin exerts a strong influence on the accumulation of glycosylated quercetin forms. Such variability is not limited to these cultivars. Wines from Garnacha (Spain) and Aglianico (Lucania region) also display differences in both total quercetin and glycoside content, underscoring the combined effects of varietal genetics, terroir, and winemaking practices. Differences in vintage further contribute to the observed variability, suggesting that climatic conditions during berry development may modulate glycosyltransferase activity and consequently influence the glycoside profile.

The analysed dataset (Table S1) includes many values for quercetin and its glycosides in wines, spanning vintages from 1982 to the present day. However, while it is not possible to draw clear conclusions about the impact of factors such as variety for quercetin aglycone, as was clearly observed after applying the Kruskal–Wallis analysis (Fig. 3), a significant impact of variety was detected for quercetin glycosides with respect to Cabernet Sauvignon and Pinot Noir for Aglianico and Sangiovese, as shown in Fig. 4. Factors such as the year of vintage or geographical origin do not affect the content of these flavonols in wines. The dataset's heterogeneity, primarily due to the wide range of wine ages at the time of analysis and the lack of standardised methods across studies, prevents meaningful comparisons. Therefore, it is not possible to evaluate the effect of a single variable given the numerous confounding factors involved.

## 5 Factors affecting content in grapes, extraction and evolution of flavonols during red grape winemaking

Flavonol glycosides are predominantly localised in the epidermal (outermost) and hypodermal (inner, thick-walled) layers of grape skins. Consequently, viticultural factors play a crucial role in modulating quercetin synthesis in grape berries, with light exposure being the most influential factor. Due to their localisation within the berry, maceration significantly enhances the extraction of these phenolic compounds into grape must and, subsequently, into wine.This highlights the importance of the maceration process in determining the final flavonol composition of the wine. Even if white grape varieties generally have lower flavonol concentrations than red cultivars (Mattivi et al., 2006), also in this case skin maceration in white winemaking has been shown to increase quercetin and its glycosides levels in the resulting wines (Ramey et al., 1986). However, since conventional white winemaking generally omits the maceration step, quercetin precipitation is uncommon and not considered a significant cause of instability in white wines. Red wines are more vulnerable to quercetin instability, primarily due to the prolonged contact of the must with grape skins and seeds happening during maceration/fermentation phases. Additionally, red grape cultivars display significantly higher variability in the levels of quercetin and kaempferol derivatives compared to white varieties. This enhanced chemical diversity is plausibly linked to the presence of an additional myricetin branch within the flavonol biosynthesis pathway, contributing to a more extensive repertoire of flavonol metabolites (Liang et al., 2012).

**Fig. 5:**
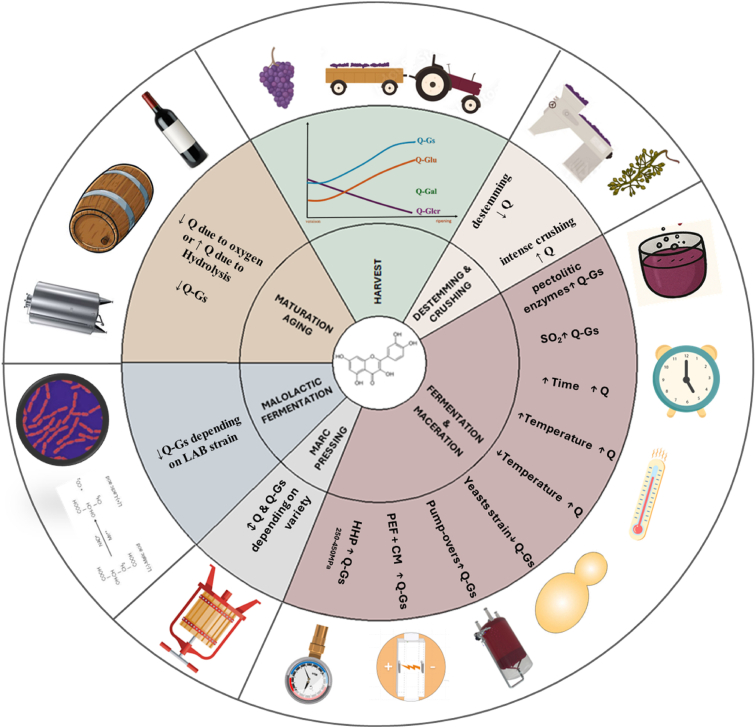
Factors affecting the concentration of flavonols during the winemaking process. ∗Q: quercetin aglycone; Q-gs: quercetin glycosides; Q-glu: quercetin-glucosides; Q-gal: quercetin-galactoside; Q-Glcr: quercetin glucuronide; PEF (pulsed electric field); CM (cold maceration); HHP (high hydrostatic pressure); LAB (lactic acid bacteria).

A multitude of oenological parameters specific to red wine production dictate flavonol composition and subsequent potential for instability. This influence begins with the precise timing of the grape harvest. The following section will systematically analyse the viticultural factors affecting flavonols accumulation in grape berries and critical phases of the vinification process for red wines. The focus will be on the mechanisms of flavonol extraction and subsequent transformation during winemaking, and their implications for the physicochemical stability of the finished wine (Fig. 5).

### 5.1 Viticultural practices

Viticultural factors play a crucial role in modulating quercetin synthesis in grape berries, with light exposure being the most influential factor. The biosynthesis of quercetin is closely linked to solar radiation, particularly UV-B wavelengths as these compounds play a critical defensive role in the grape berries by serving as photoprotective agents against UV radiation and lipid peroxidation (Rienth et al., 2021).

Vineyard management techniques that modify canopy architecture, such as leaf removal and shoot positioning, have a direct impact on cluster light interception and berry microclimate. This, in turn, influences flavonol content. Haselgrove et al. (2000) found that Shiraz berries exposed to higher levels of sunlight had higher concentrations of quercetin-3-glucoside and lower levels of malvidin-3-glucoside. Throughout all sampling stages, clusters exposed to sunlight consistently showed significantly higher levels of quercetin-3-glucoside per berry than shaded clusters. This finding confirmes that of Price et al. (1995) regarding Pinot noir. For instance, Pinot noir berries that were exposed to full sunlight accumulated approximately ten times more quercetin-*O*-glucoside than berries that were grown in shaded conditions. In line with these findings, minimal cluster exposure (e.g. absence of leaf removal) has been linked to reduced flavonol glycoside synthesis (Downey et al., 2004), whereas cultural practices that increase sunlight exposure (e.g. leaf removal) have been shown to significantly enhance the content of extractable glycosidic flavonols in grapes. This has been demonstrated in studies on Merlot (Spayd et al., 2002) and Sangiovese (Lanati et al., 2014). Basal defoliation increased quercetin-3-*O*-glucoside by 46 %, quercetin-3-*O*-glucuronide by 185 %, and kaempferol by 297 % compared to undefoliated vines in Sangiovese; similarly, defoliated Merlot vines showed a three-to four-fold increase in quercetins and kaempferol (Giordano et al., 2025). Concerning the timing of light exposure, Lanati et al. (2014) found that performing early leaf removal (pre-flowering) led to a greater increase in flavonol synthesis compared to later removal (at veraison). Recently also Poni et al. (2023) showed that early leaf removal was more effective than defoliation at veraison in promoting total flavonol accumulation. In addition, Li et al., (2022) (2022) reported that using photoselective nets reduced flavonol content by 20 %–30 % compared to control due to their effects on decreasing the exposure of vines to UV-B radiation. These results further underscore the importance of reporting not only the intensity and spectral characteristics of shading but also the specific timing of application.

The physical arrangement of the canopy affects light interception and, consequently, flavonol accumulation. In Cabernet Sauvignon vines under a fan training system, clusters exposed to light had significantly higher flavonol concentrations than shaded clusters. Wines produced from grapes originating from the upper zones of the cluster (which receive sufficient light) generally have higher concentrations of monomeric anthocyanins and flavonols than those from the lower zones (Tian et al., 2022). Additionally, Yu et al. (2024) demonstrated that the espalier training system enhances the total flavonol content of Guipu No. 6, a powdery mildew-tolerant variety, surpassing the trellis system.

The irrigation regime can further modulate flavonol accumulation, often indirectly, by influencing vine vigour and cluster exposure; studies on Monastrell grapes under semiarid conditions revealed that a non-irrigated regime resulted in higher contents of total flavonols compared to regulated irrigation deficit (Pérez-Álvarez et al., 2021). Severe deficit irrigation applied before veraison dramatically enhanced total flavonol accumulation even in Sangiovese berries, increasing content by up to 126 % compared to fully irrigated controls (Palai et al., 2022). This increase is often attributed indirectly to water deficit effects, which reduce vegetative growth, leading to less cluster shading and thus greater sun exposure, enhancing flavonoid synthesis. Compared to fully irrigated conditions, moderate water deficit determined increased concentrations of quercetin and related flavonol glycosides (Chaves et al., 2010; Pérez-Álvarez et al., 2021). However, the magnitude and direction of the effects of irrigation remain highly dependent on cultivar, environmental conditions, and vineyard management, highlighting the complex interaction between water availability and light exposure in determining the final composition of flavonols.

Several modern viticultural tools modulate flavonol biosynthesis. New bio-protectors (based on beta-carotene and alpha-pinene), applied at veraison, consistently and significantly reduced flavonol accumulation across all tested cultivars (Sangiovese, Trebbiano Toscano, Grillo, Carricante, Merlot, Grenache). In Sangiovese, the application of this bioprotector significantly decreased quercetin-3-O-glucoside (−13 %), quercetin-3-*O*-glucuronide (−22 %), and kaempferol (−20 %) in undefoliated vines (Giordano et al., 2025). In contrast, foliar application of kaolin particle film mitigates heat stress, resulting in a stimulatory effect on the flavonoid pathway and an increase in quercetin and rutin in Touriga Nacional (Conde et al., 2016). In an experiment in which Sangiovese vineyards were treated with soil application of zeolite, compost, and a mixture of zeolite and compost made from the reuse of grape processing waste, a lower accumulation of quercetin glycosides was detected in grapes treated with zeolite and with mixture (Cataldo et al., 2023).

Viticultural practices operate along a complex physiological continuum: techniques that maximize direct light exposure (like basal leaf removal or severe deficit irrigation leading to canopy breakdown) dramatically increase the production of photoprotective flavonols, including quercetin glycosides. However, mitigation strategies (like applying specific bioprotectors or improving soil water capacity with zeolite) reduce this biosynthesis.

### 5.2 Harvest

The timing of the harvest is one of the most critical decisions in winemaking depending on biosynthesis and accumulation of specific metabolites in grapes. Downey et al. (2003) showed that during grape berry ripening the concentration curve for the flavonol aglycones was distinctly non-linear, and there were two different stages over berry development: a first stage around flowering, and a second stage in which concentrations rose rapidly in the last phases of ripening (Downey et al., 2004). In the same study, the authors showed that on a per berry bases glycosides and flavonols increased from fruit set trough veraison towards harvest. Giovanelli and Brenna, (2007) observed similar patterns across multiple cultivars, reporting maximum flavonols (as the sum of rutin, quercetin and quercetin-3-*O*-glucoside) levels before veraison, followed by a general decline during first phases of ripening and a further increase at maturity. In the study of Fang et al. (2013), carried out on Cabernet Sauvignon, authors confirmed that the total flavonol content had two accumulation peaks during the whole grape berry development, one appeared at the rapid growth phase, the other occurred at the ripening period, and then remained at a high level in the ripe fruit. They also observed that at the late stage of the lag growth phase, almost no free flavonol was detected. In Crimson Seedless grape Brar et al. (2008) observed that for two consecutive years the concentration of quercetin 3-*O*-glucoside in the berry skin increased during berry development and ripening while the concentration of quercetin 3-*O*-glucuronide in the berry skin decreased during the same period. Throughout ripening and over ripening of Cabernet Sauvignon Martínez-Lüscher et al. (2019) found also that changes in flavonol profile displayed different patterns among the three major groups of flavonols. For instance, the proportion of quercetin and myricetin flavonols combined was always more than 80 % of total flavonols with quercetin starting around the 85 % of all flavonols at verison and therefore, the increase in the proportion of one, was accompanied by a decrease in the other. The proportion of quercetin decreased in all the treatments until harvest and increasing shading factors resulted in lower quercetin proportions. During ripening quercetin glycosides trend in three red grape cultivars grown in China (Cabernet Sauvignon, Merlot and Cabernet Gernischt) showed a great variability: quercetin-3-*O*-glucoside for Merlot and Cabernet Sauvignon increased, quercetin-3-*O*-glucuronide decreased with different rates depending on the variety while quercetin-3-*O*-galactoside was stable and decreased only in the case of Merlot in 2010 (Liang et al., 2012). In the study of Tavernier et al., (2025), a decreasing trend along berry development was detected for quercetin-3-*O*-glucuronide. Authors also found an accumulation of flavonols (kaempferol, quercetin, isorhamnetin, and myricetin derivatives) late in berry ripening.

The ultimate flavonol profile of wine depends not only on the initial concentration of these compounds in the grape berry, but also on their extraction efficiency and transformation reactions during vinification, especially for grapes harvested at different levels of ripeness. The impact of harvest maturity on flavonol transfer was investigated in a study involving Aglianico grapes harvested across a gradient of soluble solids (°Brix) levels. The study examined the correlation between harvest maturity and the final quercetin concentration in the resultant wines. The data showed that an increase in °Brix at harvest did not lead to a statistically significant change in quercetin levels in the wine. The sole exception was a minor yet significant lower level observed in wines derived from overripe grapes (Gambuti et al., 2007; Genovese et al., 2021). This finding suggests that, in addition to the biosynthesis and accumulation of quercetin within the berry skins, the extractability coefficient of the compound from the skin matrix must also be considered a critical determinant of its final concentration in the wine. However, the potential adsorption of quercetin aglycone and glycosides onto the solid matrix of pomace during maceration and fermentation must be considered, as this can diminish the net transfer to the liquid phase (Errichiello et al., 2024).

### 5.3 Destemming and crushing

Although destemming is a common protocol in red winemaking, the decision to retain or remove the stems during maceration is often influenced by the characteristics of the cultivar and the specific oenological objectives. Studies have identified quercetin-3-O-glucuronide as the predominant phenolic compound extracted from the stems, followed by catechin and dihydroquercetin-3-O- rhamnoside (astilbin) (Goufo et al., 2020; Souquet et al., 2000). The inadvertent presence of leaf material during must preparation has also been linked to excessive quercetin uptake (Jackson, 2020). The impact of elevated leaf content is significant enough to induce quercetin precipitation and subsequent deposit formation, particularly in white wines (Ziemelis et al., 1982). These observations suggest that pre-crush removal of stems, leaves and stalks offers clear advantages in terms of flavonol management and stability. Conversely, vinification protocols that deliberately incorporate stems, such as carbonic maceration or whole-bunch fermentation, should logically result in elevated quercetin concentrations in the finished wine. However, limited data exist regarding the total flavonol content under these conditions. At our knowledge only in one study Shmigelskaya et al. (2021) found that carbonic maceration led to an accumulation of quercetin and quercetin-3-*O*-glycosides at a concentration almost double that achieved through standard vinification techniques.

Whether preceded by destemming or not, crushing serves to rupture the berry skin and release the juice, thereby initiating the fermentation phase. The efficiency of phenolic extraction is linked to the structural integrity and chemical composition of grape cell walls. The intensity of the crushing action correlates directly with the extent of disruption of skin cell walls, consequently enhancing the extraction yield of phenolic compounds. Since flavonol glycosides are predominantly found in the upper layers of the grape skin epidermis (Downey et al., 2003), they are readily transferred into the must at this early stage of winemaking. As with the impact observed for stems, it is obvious that a more aggressive mechanical crushing will result in a higher final concentration of quercetin glycosides being released into the wine.

### 5.4 Alcoholic fermentation/Maceration

Glycosylated flavonols are found in the same vacuoles as anthocyanins in grape berries, specifically within the subepidermal cells of the skin (Cheynier and Rigaud, 1986). Their release into the must mainly occurs during maceration and fermentation. This extraction process is influenced by several factors, including maceration time, fermentation temperature, the yeast strain and the progressive increase in ethanol concentration, which enhances cell wall permeability and promotes the solubilization of phenolic compounds (Fougère et al., 2023). Gambuti et al. (2009) reported that anthocyanin extraction reaches its maximum around the fourth day of maceration. In contrast, the quercetin content increased slowly throughout the maceration period. This suggests either that quercetin glycosides are released at a slower rate than anthocyanins, or that the extracted glycosides are slowly hydrolysed during maceration. Alencar et al. (2018) reported no specific trend for isorhamnetin-3-*O*-glucoside, quercetin-3-*O*-glucoside, kaempferol-3-*O*-glucoside and rutin. Conversely, rutin levels were found to be higher in long-macerated wines, suggesting that its extraction is favoured by prolonged skin contact and the solvent effect of ethanol (Gambuti et al., 2007). Koyama et al. (2007) reported that by day 10 of maceration the extraction curves showed an overall decrease of 32 % from the peak of extraction of flavonols for different winemaking treatments, including cold soak and post-fermentative heating at 42 °C. However, in cold musts, the maximum extraction of quercetin occurred later than that of anthocyanins. Gambuti et al. (2004) also observed an increase in quercetin concentration during maceration, which supports earlier findings by Mérida et al. (1991), who demonstrated a positive correlation between maceration length and quercetin extraction efficiency. In the same study, Mérida et al. (1991) reported that temperature did not significantly affect the extraction of phenolic compounds during the first 16 h of maceration. After 48 h, a significant effect on quercetin concentration was observed when the temperature increased from 10 °C to 25 °C. It is well established that enhanced phenolic extraction at higher fermentation temperatures is due to increased hypodermal cell permeability, which promotes the release of anthocyanins and increases the solubility of other phenolic compounds in the wine matrix. Ramey et al. (1986) also confirmed the strong temperature dependency of flavonol extraction in Chardonnay wine.

Winemaking techniques that focus on the extraction of phenolic compounds during maceration are often employed. Examples include pre-fermentation cold maceration, high hydrostatic pressure (HHP) and pulsed electric fields (PEF). María Jesús Cejudo-Bastante et al. (2014) observed that flavonols were more easily extracted using pre-fermentative cold maceration. This study involved two periods of skin maceration at the same temperature (5–11 °C): eight days (SCM, short cold maceration time) and 12 days (LCM, long cold maceration time). The results showed that long cold maceration results in higher flavonol extraction than traditional maceration or short cold maceration. In another study, applying PEF (pulsed electric field) during cold maceration of Cabernet Sauvignon and Cabernet Franc improved quercetin-3-O-glucoside extraction by 100 % and 74 %, respectively, compared to the control (Vejarano and Luján-Corro, 2022.). High hydrostatic pressure (HHP) is a batch processing technology that can enhance the extraction of phenolics into must and wine, as well as lowering microbial populations and preserving volatile compounds (Vejarano et al., 2022). Moderate pressures (250–450 MPa) increased flavonol content over time, whereas very high pressure (650 MPa) did not; this is likely since excessive pressure promotes the oxidative degradation of polyphenols during maceration (Otero et al., 2025).

Another key enological practice that influences flavonol extraction is cap management. This refers to how the solid fraction (skins, seeds and stems) that float or becomes compacted at the top of the fermenting must is handled during fermentation and maceration. Techniques such as punch-downs, pump-overs and submerging or leaving the cap undisturbed can significantly affect the extraction, distribution and preservation of phenolic compounds. Fisher et al. (2000) investigated the impact of different cap management techniques and found that pump-overs consistently resulted in the highest quercetin concentrations. They attributed this effect to enhanced skin maceration through mechanical movements, as well as the influence of β-glucosidase activity. The use of pectolytic enzymes, sulphur dioxide levels and pH conditions can also affect the extraction and stability of flavonols during winemaking. Using pectolytic enzymes and SO_2_ has been shown to increase flavonol levels in wine, such as those of quercetin and rutin, likely due to enhanced extraction from grape skins and the partial hydrolysis of glycosidic precursors (Gambuti et al., 2007). However, the purity of commercial enzyme preparations must be considered, as they may contain glycosidase side activities that can hydrolyse flavonol glycosides and release the corresponding aglycone forms. This unintended activity could partly explain the higher concentrations of free quercetin observed in wines produced using enzymes. Therefore, when evaluating the effect of pectolytic enzymes on flavonol extraction, it is essential to distinguish between genuine increases in extraction efficiency and potential contributions from glycosidic hydrolysis due to enzymatic impurities.

Regarding the type of fermentation vessel, Fang et al. (2008) reported that, under comparable vinification conditions, the use of new wooden barrel, revolving pot and the stainless-steel tank did not markedly affect flavonol extraction. The analysis revealed that only myricetin was present at higher levels in Cabernet Sauvignon wines fermented in new wooden barrel or in a revolving pot with respect to stainless-steel tank.

Yeast strains responsible for fermentation can significantly impact the extraction and transformation of flavonols, including quercetin and its glycosides. Enzymes originating from grapes and microorganisms promote the hydrolysis of phenolic compounds during winemaking (Villena et al., 2007). The available evidence indicates that non-*Saccharomyces* species generally exhibit higher β-glucosidase activity compared with *Saccharomyces cerevisiae*. Among *Saccharomyces. cerevisiae*, only a few strains display significant extracellular activity (Zhang et al., 2021). In addition, the effectiveness of this enzymatic pathway is not solely strain-dependent but is also strongly influenced by must composition and fermentation conditions, including glucose and ethanol concentrations, pH, temperature, and sulphur dioxide levels. (Zhang et al., 2021). Mixed or sequential inoculation strategies have generally not been associated with significant changes in quercetin accumulation compared to single *Saccharomyces cerevisiae* fermentations. However, Romboli et al. (2015) reported that fermentation with *Saccharomyces cerevisiae* alone resulted in a decrease in quercetin in Sangiovese wines, whereas sequential inoculation with *C. zemplinina* followed by *S. cerevisiae* led to a greater reduction in quercetin-3-*O*-galactoside and quercetin-3-*O*-glucuronide. This effect was attributed to the higher *β*-glucosidase activity of *C. zemplinina*, which may have enhanced the hydrolysis of flavonol glycosides.

Yeast strains also exhibit considerable variability in their ability to adsorb flavonols during fermentation. Rizzo et al. (2006) reported that among 23 *S. cerevisiae* strains, Sc1483 showed the highest capacity to adsorb rutin. Similarly, Jagatić Korenika et al. (2021) observed that quercetin-3-O-glucoside concentrations differed significantly among wines fermented with different yeast strains. Notably, quercetin levels were strongly influenced by yeast only in one of the wines examined in their study, whereas kaempferol concentrations appeared unaffected by the yeast strains used.

### 5.5 Malolactic fermentation

In addition to alcoholic fermentation, the production of red wine typically involves malolactic fermentation (MLF), followed by ageing in barrels or bottles. Hernández et al. (2006) evaluated the impact of MLF performed in barrels versus stainless steel tank, reporting that quercetin aglycone levels were unaffected. However, quercetin glycosides exhibited different behaviours: quercetin-glucuronide was absent in wines undergoing barrel MLF, whereas quercetin-galactoside exhibited only a slight decrease in stainless steel fermentations. In a subsequent study (Hernández et al., 2007), it was demonstrated that the concentration of free flavonols may depend on the strain of lactic acid bacteria (LAB) conducting MLF. Increases in aglycones such as myricetin and quercetin were observed during both spontaneous fermentations and in wines inoculated with a *Oenococcus oeni* strain (Oe-18), thus suggests a possible β-glycosidasic activity of specific LAB.

### 5.6 Marc pressing

After maceration, the wine is separated into two main fractions: free-run wine, which drains from the pomace by gravity, and press wine, which is mechanically extracted from the remaining solids. The influence of marc pressing on the final flavonol profile is cultivar-dependent and not consistently uniform. Higher total flavonol concentrations have been reported in the free-run wines of Sangiovese (Rinaldi et al., 2020) and in Aglianico wines blended with only 7 % of pressed wine (Gambuti et al., 2007). In these cases, the free-run fractions showed elevated levels of quercetin and its conjugated derivatives. In contrast, data for Piedirosso and Nerello Mascalese wines showed that pressing the marc increased the final quercetin content in the blended wines (Gambuti et al., 2004). This variability suggests a complex interplay between extraction and other physicochemical phenomena. For example, it is necessary to consider that quercetin is one of the most abundant flavonoid compounds recoverable from red pomace (Errichiello et al., 2024). Notably, Careri et al. (2003) detected a quercetin content in wine pomace that was more than two orders of magnitude higher than the initial concentration in the grape skins. This strongly suggests a significant adsorption effect onto the grape skins during fermentation. Thus, although flavonols are primarily concentrated in grape skins (Makris et al., 2006), mechanically pressing the grape marc does not necessarily lead to greater extraction into the wine. This apparent paradox is likely due to the grape marc being highly enriched in polysaccharides and soluble pectins that can interact with and retain flavonols, thereby reducing their solubility and extractability (Chirug et al., 2018). As the composition and concentration of these macromolecules are cultivar-dependent, the grape variety plays a crucial role in determining the efficacy of pressing for flavonol recovery.

### 5.7 Maturation/aging

Just after fermentation ends flavonols and related glycosides are involved in two major groups of reactions: the hydrolysis of glycosides and interactions with other phenolic compounds, mainly mediated by dissolved oxygen. However, for flavonols aglycones, a general and rapid decrease is observed in the first months after malolactic fermentation (Gil-Muñoz et al., 1999). Aglycones can degrade upon exposure to heat, enzymes, and oxidative chemical species such as free radicals (Makris and Rossiter, 2002), as well as being involved in the formation of high-molecular-mass polymeric polyphenols (Suo et al., 2019). Concerning glycosides, numerous studies have shown that flavonol glycosides undergo hydrolysis and decrease in red wines over the first few years of storage, whereas aglycones increase. In a study on Sangiovese wines, the loss after 23 months of aging was around 50 % of the initial value for almost all analysed wines, and it only reached 75 % in one case. (Gambuti et al., 2020). A similar decline in flavonol glycosides (25 %), probably due to hydrolysis, was previously shown over a period of seven months (Zafrilla et al., 2003). Obreque et al., (2023), who investigated how phenolic compounds evolve during the ageing of red wines made from six different grape varieties, found that myricetin disappeared from all samples in the early ageing. Quercetin-3-O-glucuronide exhibited a distinct varietal-dependent trend: it was not detected in Carménère prior to wood ageing but appeared after 12 months of storage. In contrast, in the other five varieties, it decreased to 0 mg/L after just six months. Quercetin-3-O-galactoside followed a different pattern. Although initially present in all varieties, it declined to undetectable levels after nine months in the barrel in some cases, while remained detectable in others even after 12 months. These results emphasise the significant varietal dependence of flavonol evolution during ageing, implying that various quercetin glycosides undergo hydrolysis at different rates.

Concerning the oxidation over time it is known that flavonols are easily oxidised (Jørgensen et al., 1998). In a previous trial, four successive air saturations, each supplying the wine with almost 6 mg/L of oxygen, showed that quercetin decreased between 65 % and 80 % of the initial value. Under real conditions, Castellari et al. (2000) demonstrated that quercetin levels decreased by over 50 % within 6 months due to oxygen supplementation during storage. These data suggest that micro-oxygenation or barrel ageing could reduce quercetin concentrations below their solubility threshold.

The type of container used for ageing wine has a significant impact on the evolution of all phenolic compounds, and the use of specific containers (wood, stainless steel or glass bottles) is now a key step in the production process. Wood containers usually used as oak heartwood does not contain flavonoid compounds, only the less used cherry wood has been shown to contain quercetin (Martinez-Gil *et al.*, 2020). Castellari et al. (2001) investigated the effects of different types of wood (oak and chestnut) and barrel sizes (225 L and 1000 L) on Sangiovese wine aged at two temperatures (12 °C and 22 °C) for 320 days. Their results showed that ageing in wooden containers compared to stainless steel led to an increase in quercetin levels, probably due to the concurrent hydrolysis of glycosides. Several studies have reported a general decrease in flavanol glycosides and aglycones after 12 months of ageing, suggesting that glycosides undergo significant acid hydrolysis during oak maturation. However, this does not necessarily result in a corresponding increase in the aglycone fraction, probably because flavonols are simultaneously involved in oxidation and condensation reactions or become insoluble in the wine matrix (Feng et al., 2024). The findings concerning quercetin glycosides are consistent with those of Fang et al. (2008), who investigated the evolution of various flavonols and their glycosides in wines aged for 240 days in 225 L and 50 L barrels that had previously held wine. Levels of quercetin in both types of barrels increased over time due to glycoside hydrolysis, with similar levels observed in both types of barrels. Additionally, the decrease in quercitrin concentrations was similar in the two types of oak barrel, while greater changes in rutin concentrations were observed in the 50 L oak barrels. In a study (Hernández et al., 2007**)** on Tempranillo wines aged in oak barrels and subsequently stored in bottles for 12 and 24 months the authors showed that the aglycone quercetin showed different behaviour depending on the oak wood used in the previous aging phase: the greatest concentrations of flavonols (quercetin and its glycosides, myricetin, and its glucoside) were found in wines previously aged in Spanish *Quercus petraea*, followed by Spanish *Q. pyrenaica* and American *Q. albus.* They also found in bottle a decrease of quercetin-3-glucuronide and disappearance of quercetin-3-galactoside between 12 and 24 months of aging.

As expected, the effects observed when wines were in contact with wood, mainly linked to the hydrolysis glycosides and degradation of quercetin (Monagas et al., 2005), were less evident when wood ageing was compared to bottle ageing. This is likely due to lower dissolved oxygen levels over the ageing period, flavonol adsorption onto the container surface, and the possible easier induction of quercetin crystallisation in barrel.

## 6 Oenological strategies to counteract phenolic instability

Of the various proven strategies, the use of PVPP (Lanati et al., 2014; Vendramin et al., 2022; Borghini 2022; Picariello et al., 2023) has consistently resulted in a decrease of quercetin in wine, showing a direct correlation between the reduction in quercetin levels and the increase in the PVPP dose used (Table 2). In 1990, the first references appeared demonstrating the use of PVPP, a synthetic polymer (polyvinylpolypyrrolidone) for removing quercetin. At that time, the presence of quercetin aglycone in red wines was associated with the mutagenic activity of wines (Rueff et al., 1990). As a result, numerous strategies were tested to eliminate this flavonol from red wines to reduce their mutagenic potential. In the same year, the application of PVPP on 38 red wines resulted in an average reduction of quercetin by 90 %, highlighting a significant affinity between this polymer and quercetin (Fluss et al., 1990). In 2006, following the appearance of this new instability in white wines Laborde et al. (2006) initiated a molecular study to investigate the interaction between quercetin and synthetic polymers such as PVPP. The results, both in white wine and methanol solution, demonstrated that PVPP exhibited a higher affinity for the aglycone form and a lower affinity for quercetin 3-*O*-glucoside owing to the formation of the complex thanks to the hydrophobic interaction between the phenolic rings of quercetin and pyrrolidinone rings as well as to the establishment of H bonds between the hydroxyl functions of quercetin and the CO-N linkages of PVPP. We should consider that a noticeable impact on colour and polyphenol adsorption was observed after the use of PVPP at high dose. Even other compounds, such as thiols aroma demonstrated specific affinities for PVPP. (Gil et al., 2019). However, the use of PVPP is not permitted in the production of organic wines (EU regulation. n. 203/2012), therefore numerous alternative products and strategies have been proposed over the years. Lanati et al. (2014) investigated potential solutions to the issue by testing not only the effects of PVPP but also of activated carbon at two different doses and two different practices of oxygenation (air saturation and wine racking). The results demonstrated that all these practices could decrease flavonoid content, particularly quercetin and their glycosides, in wines. Authors observed that the quality was slightly affected: the treatments with PVPP and carbon especially at high doses, resulted in a maximum removal of monomeric anthocyanins of 5.06 % with respect to control wines, thus preserving red wine colour. However, as the author suggests, this study was conducted using aged wines, where most of the anthocyanins are in a polymerized form. Therefore, it would be important to assess the effects of these treatments on younger wines, where the anthocyanins are mainly monomeric and could be more easily removed, potentially leading to a loss of colour. Exposing the wine to oxygen during the maturation process has proven to be the most effective way to remove quercetin aglycone and glycosides. Also, Gambuti et al. (2020) suggests that techniques like micro-oxygenation and aging in wooden barrels may reduce the risk of quercetin precipitation in finished wines.

**Table 2:** Oenological Strategies to counteract quercetin instability.

	Additives/Practices:	Doses:	Effects on quercetin:	Effects on quercetin-glycosides:	References:
Addition of oenological additives	PVPP	60 mg/100 mL	63–95 %		Fluss et al., (1990)

Effect of enzymes	Commercial enzymes	their highest recommended dosage for wine		Some enzymes contain glucuronidase activity	Wightman et al., (1997)

Addition of oenological additives	PVPP	various concentrations of PVPP (maximum: 80 g/hL)	strong association with quercetin		Laborde et al., (2006)

Addition of oenological additives	PVPP	5 g/hL	−5.86 %	−25.8 %	Lanati et al., (2014)
	PVPP	10 g/hL	−59.3 %	−6.82 %	
	Carbon	5 g/hL	−16.7 %	−35.4 %	
	Carbon	10 g/hL	−55.3 %	−15.1 %	
Oxygenation	Oxygenation	3 pour on 100 mL of sample every 3 days for 10 days.	−9.93 %	−35 %	Lanati et al., (2014)
Oxygenation	MicroOxygenation	to 100 ml of wine by insufflating air trought a syringe 3 times every 3 days for 10 days	−61.2 %	−49 %	

Oxidation effect on Quercetin	four consecutive air saturation cycles	saturated with air until the oxygen level of the wine reached 6,6 mg/L	6.6 mg/L of oxygen turned out to be enough to decrease by more than 50 % the initial amount of quercetin	slight decrease of quercetin glycosides occurred	Gambuti et al., (2020)

Addition of oenological additives	PVPP	20 g/hL	direct correspondence between decrease of Q and the increase in the PVPP dose used	not statistically significant	Vendramin et al., (2022)
	PVPP	40 g/hL		not statistically significant	
	PVPP	60 g/hL		not statistically significant	
	Vegetal Protein (potato protein)	20 g/hL	did not show any effect	not statistically significant	
	Sodic bentonite	20 g/hL	did not show any effect	not statistically significant	
	PVP/PVI polymer	20 g/hL	not statistically significant	not statistically significant	
	PVP/PVI polymer	60 g/hL	significant effect only at this dose	not statistically significant	
Effect of enzymes	Experimental pectolytic enzyme with secondary glycosidase activity	4 g/hL	increase in the aglycone (+261 % after 7 days of treatments)	stark decrease in the quercetin-3-glucoside concentration (−18 % after 7 days of treatments)	

	Additives/Practices:	Doses:	Effects on Q:	Effects on Q-Gs:	References:
Effect of enzymes	β-glucanases enzymes			had no effect	Borghini (2022)
	Pectolytic enzymes			increase in quercetin aglycone, with a corresponding decrease in the glucoside fraction and especially the glucuronide.	
	β-glucosidases enzymes				
	a mixture of pectolytic enzymes and α e β-glucosidase enzymes				
Addition of oenological additives	PVPP		considerable reduction in aglycone		
	vegetable carbons		considerable reduction in aglycone, the second carbon, completely removed the concentration of aglycone.		
Addition of oenological additives	PVPP	0.2; 0.5; 0.8; 1.2; 1.8 g/L	Best adsorbent (−80 %), demonstrated a clear interaction with quercetin aglycone		Picariello et al., (2023)
	Yeast Lysate (YL1, YL2, YL3, YL4)	0.1; 0.2; 0.3; 0.4; 0.5 g/L	YLs greatly differed for the adsorption capability, L4 showing the better removal properties.		
	Potato Protein	0.05; 0.1; 0.3; 0.5; 0.7; 1 g/L	Until 20 % removal	Not very effective	
	MIX (PVPP, vegetable proteins and YL4)	0.2; 0.5; 0.8; 1.2; 1.8 g/L	efficiently used to decrease quercetin	efficiently used to remove to decrease quercetin glycosides	
	MIX (PVPP, vegetable proteins and YL4) on 11 Sangiovese wines	0.4 g/L	drastically decreases the quercetin concentration in wines (from 26,69 % to 39,05 %)	No sharp decrease was observed for quercetin glycosides, except for samples W9 and W11.	
Addition of nucleation seeds	quercetin powder as nucleation seeds	around 2.07 mmol/L	substantial reduction of over 75 % of the initial Q value was observed at 2 °C	efficiently used to remove quercetin glycosides	Luciano et al., (2024)

In addition, the aglycone is only one aspect of the problem, it is also necessary to consider the ongoing hydrolysis of glycosides during the wine evolution (Table 2). These strategies include the use of enzymes that rapidly reduce the glycosidic fraction of quercetin and release the aglycon fraction, facilitating its precipitation before bottling (Vendramin et al., 2022). As early as 1997, some enzymes used in winemaking process were found to possess secondary glucuronidase activity (Wightman et *al.,*1997). This finding is significant because a substantial portion of glycosides in wine may consist of quercetin glucuronide (Table S1). The identification of enzymes with this secondary activity is crucial, as they could potentially aid in breaking down these glycosides. An important consideration when using enzymes is, also in this case, the potential loss of colour, as enzymes can promote the hydrolysis of anthocyanins, potentially reducing colour stability. Vendramin study shows also that the use of enzymes caused only slight modifications in colour intensity. Additionally, yeast lysates have been also explored, yielding promising results for a specific lysate (Picariello et al., 2023) and further research are needed to understand the mechanism involved in the possible quercetin adsorption. In this study, Picariello et al. (2023) evaluated also the effectiveness of a vegetable protein as a fining agent. The results indicated that the vegetable protein had only a minimal effect on quercetin adsorption and removal. Promising results have also been observed with the use of a mixture composed of PVPP, vegetable protein, and the yeast lysate that was shown to adsorb quercetin. All these findings highlighted the application of each additive or fining agent must be accompanied by a thorough evaluation of its potential impact on both aroma and colour, to ensure that the overall quality of the wine is maintained.

However, the addition of agents to remove quercetin and quercetin glycosides from wine is only one possible strategy, recently Luciano et al. (2024) highlights another important finding that could help to find a solution by accelerating quercetin precipitation. Authors showed that the addition of nucleation seeds, such as quercetin powder, facilitates the rapid precipitation of quercetin in solution at lower temperatures. This suggests a potential strategy to accelerate precipitation and thus limit quercetin precipitation in bottled wines. However, further research is necessary to assess the impact on other wine components and to identify the best practices for industrial application.

Another possible approach is to find the factors and additives increasing a solubility. At our knowledge until now this approach has not been explored. A further promising concept for future oenological applications is the use of a molecularly imprinted polymer capable of selectively adsorbing quercetin and its glycosides. This technique has been studied for the potential extraction of valuable compounds from diatomaceous earth used in the wine filtration process or winemaking residues (Gomes et al., 2024). Although this seems an interesting approach the economical and regulation issue should limit their applicability.

## 7 New analytical tools to forecast wine instability

Recent advances in machine learning and deep learning have significantly improved the ability to predict parameters that are key to wine stability and quality assurance (Ranaweera et al., 2021a, 2024). Using high-dimensional datasets allows computational models to predict indicators of wine quality and ageing-related instability.

The earliest applications of chemometric modelling in oenology emerged in the early 2000s. The use of partial least squares regression (PLSR) in conjunction with high-performance liquid chromatography (HPLC), near-infrared (NIR) spectroscopy (Cozzolino et al., 2004) and UV–Vis spectral data (Skogerson et al., 2007) produced robust calibration statistics for predicting major phenolic compound groups. However, it has only been in recent years that the prediction of specific phenolic compounds, such as quercetin and its glycosides, has become accurate, primarily using the absorbance-transmission and fluorescence excitation-emission matrix (A-TEEM) technique combined with multivariate calibration. Ranaweera et al. (2021) analysed Shiraz, Cabernet Sauvignon and Merlot wines from ten Australian regions using A-TEEM on diluted samples, coupled with Extreme gradient boosting discriminant analysis (XGBDA) modelling. This approach enabled discrimination by variety and geographical origin. Similarly, Shoeber et al. (2022) demonstrated that, in Chilean Cabernet Sauvignon wines, A-TEEM integrated with an optimised regression algorithm provides robust predictions of key phenolic constituents, including quercetin, its glycosides, and additional phenolic markers relevant to precipitation risk. More recent investigations by Gilmore et al. (2022) and Armstrong et al. (2023) have further established that A-TEEM, when combined with multi-block data fusion and machine learning models (particularly XGB), is an efficient tool for the rapid assessment of incoming grape lots. These methods offer analytical speeds that surpass those of HPLC, while achieving comparable precision for phenolic quantification. Recently, in one case study concerning rosé wine storage, Temporal Convolutional Network, Long Short-Term Memory, and Gated Recurrent Unit models were used to forecast trends in parameters like dissolved oxygen, conductivity, and temperature; the Temporal Convolutional Network model demonstrated the best predictive performance for these instability indicators (Zhang *et al.*, 2025). Deep learning architectures have been also applied alongside infrared spectroscopy for the robust quantitative analysis of key components in dry red wine, including pH, total phenols, total sugars, and alcohol content, which are essential markers for wine quality assessment and stability (Chen *et al.*, 2025). The engineering of sensors could also provide an effective solution for the rapid detection of these flavonols in wine. Recently (Di Giulio et al., 2025), a molecularly imprinted polymer (MIP)-based optical sensor for the selective and sensitive detection of quercetin in red and white wines was pointed out and it may represent a promising alternative to conventional analytical methods.

## 8 Conclusions and future perspectives

### 8.1 Conclusions

The instability of flavonols represents one of the most pressing oenological challenges today for red wines. The aglycone form of quercetin is poorly soluble, favouring its precipitation as crystals and leading to the formation of undesirable deposits that compromise the visual appearance and commercial value of wines. Initially reported sporadically and mainly in varieties such as Sangiovese, this phenomenon has become increasingly common, likely due to climate change and higher solar radiation influencing flavonol biosynthesis. The complexity of the wine matrix, a colloidal system involving numerous non-covalent interactions, makes it difficult to predict and control quercetin solubility and precipitation.

A deeper understanding of the mechanisms governing the extraction, evolution and precipitation of quercetin is essential for developing targeted, sustainable winemaking protocols. Looking ahead, it will be crucial to integrate this knowledge with new analytical tools and innovative strategies, including those aimed at enhancing quercetin solubility or exploiting its interactions with other phenolic compounds.

Several strategies have been suggested to address the flavonol instability in finished wines. These include approaches aimed at removing quercetin aglycone and its glycosides below its solubility threshold by using oenological additives as PVPP, charcoal or yeast lysates, or by using specific enzymes with glycosidic activity or by micro-oxygenation to decrease the amount of free aglycon and promoting controlled precipitation prior to bottling using nucleation seeding. However, the potential side effects of each approach on colour, aroma, and overall wine stability must be carefully evaluated and none of these approaches until now used can be considered as free of collateral effect.

Looking for future studies, investigations in different areas could be considered. Here are listed the main research lines that should be more deepen in future works.

(1) Development of a rapid and simple test to predict quercetin instability in wines.

A key challenge in managing quercetin precipitation is the lack of practical, rapid analytical tools available to wineries. Although current quantification methods, mainly HPLC-UV methods, are accurate, they are also time-consuming and expensive, making them impractical for routine use during production. In addition, only the value of quercetin and glycosides are not enough to predict the future instability of wines. The development of a fast, cost-effective and user-friendly diagnostic test would enable winemakers to assess the risk of quercetin instability at an early stage of the winemaking process. Such a test could detect the concentration of free quercetin relative to its solubility threshold, for example, or measure the ratio of quercetin to other molecules such as anthocyanins and tannins, which influence its solubility. Having access to such a predictive tool would enable winemakers to make timely decisions regarding treatments (e.g. fining agents, oxygenation and enzyme use) and try to prevent deposit formation prior to bottling.

(2) Implementation of viticultural strategies and transcriptomic studies to limit flavonol production in grapes.

As flavonol biosynthesis is greatly affected by environmental factors and vineyard practices, closer collaboration between vineyard and winery management is crucial to reduce the risk of quercetin-related instability in wines. Viticultural strategies such as canopy management, controlled defoliation, modulation of cluster exposure to solar radiation and optimization of irrigation and nutrient regimes can significantly impact flavonol synthesis and accumulation in grape skins. Fine-tuning vineyard practices to balance phenolic composition may reduce flavonol accumulation to levels that do not compromise wine stability without negatively affecting quality attributes such as colour and antioxidant capacity. As the instability is linked to grape cultivar, the study of the impact of environmental factors on the expression of genes involved in quercetin and its glycosides synthesis should be also deepen by focusing on the precipitation risk in finished wines for each grape cultivar.

(3) Use of co-adjuvants or additives permitted in organic winemaking to improve quercetin stability.

In the field of winemaking practices, prospects should focus on the use of co-adjuvants that are permitted in organic wine production, such as yeast hulls or mannoproteins, which could have potential to interact with quercetin. These agents can help to solubilize quercetin affecting the colloidal equilibrium or adsorb quercetin molecules, thereby enhancing its solubility or its concentration. Unlike synthetic polymers such as PVPP whose use is restricted in organic winemaking these natural co-adjuvants offer a sustainable and regulation-compliant alternative. Further research into the mechanisms of interaction between these bio-macromolecules and quercetin, as well as optimization of their application timing and dosage, could pave the way for innovative strategies that both prevent quercetin instability and enhance overall wine quality within the framework of organic production.

(4) To study the impact of yeasts and malolactic bacteria on flavonols during winemaking.

A more focused approach on the role of microorganisms involved in fermentation on the evolution of quercetin and glycosides extracted from grapes during maceration could help to find sustainable and efficient practices to be used avoiding the risk of losing sensory and positive compounds in finished wines usually linked to the addition of additives. A particular attention should be given to the capability of microorganisms to adsorb phenolic compounds and to hydrolyse glycosides.

(5) Developing predictive models thanks to machine and deep learning technologies.

The integration of Internet of Things (IoT) monitoring with deep learning models to forecast physicochemical parameters critical for predicting potential quality deterioration during wine storage is an increasingly studied area. Thanks to these new technologies, a rapid and reliable test for evaluating the risk of flavonol precipitation is likely to be available soon. This will help ensure that the correct strategy is applied when managing quercetin and quercetin glycosides during winemaking.

## CRediT authorship contribution statement

Alessandra Luciano: Conceptualization, Data

curation, Writing – original draft.

Luigi Moio: Funding acquisition.

Supervision. Angelita Gambuti: Conceptualization, Project administration,

Writing – review & editing.

## Declaration of competing interest

The authors declare that they have no known competing financial interests or personal relationships that could have appeared to influence the work reported in this paper.
