# Supplementary material for: Quercetin instability in red wines: Insights into precipitation and control approaches
Source: Curr Res Food Sci. 2026 Jan 22;12:101319. doi: 10.1016/j.crfs.2026.101319 (PMC12918166; doi:10.1016/j.crfs.2026.101319)
Supplement: Multimedia component 1 [file mmc1.docx]

**Supplementary material (Table S1)**

**Table S1** Content of quercetin and quercetin glycosides in red wines.

| **Variety/Denomination:** | **Origin^§^:** | **Content of Quercetin:** | **Content of Glycosides^#^:** | **Vintage:** | **References:** |
| --- | --- | --- | --- | --- | --- |
| Abruzzo Doc, Montepulciano | Abruzzo, Italy | 1.03 mg/L | Quercitrin: 0.20 mg/L | 2 years | Simonetti et al., (2022) |
|  |  |  | Quercetin-3-*O*-glucoside: 0.63 mg/L |  |  |
|  |  |  | Quercetin 3-*O*-rutinoside: 0.03 mg/L |  |  |
| Agiorgitiko | Peloponnese,Greece | 8.46 mg/L |  |  | Sakkiadi et al., (2001) |
| Agiorgitiko | Peloponesse, Greece |  | Quercetin 3-*O*-rutinoside 8.2- 44.5 mg/L* | 1998 | Arnous et al., (2001) |
|  |  |  | Quercetin 3-*O*-rhamnoside: 26.0-96.2 mg/L* |  |  |
| Aglianico (4) | Lucania region,Italy | 2.1-4.1 mg/L* |  | 1997 | Pavia et al., (2001) |
| Aglianico (7) | Italy | 1.8-9.8 mg/L | 17.5- 64.5 mg/L as sum of glycosides | 2023 | Luciano et al., (2025) |
| Amarone |  | 0.35 mg/L |  |  | Lante et al., (2004) |
| Barbera | Piemonte,Italy | 4.80 mg/L | Quercitrin: 0.02 mg/L | 3 years | Simonetti et al., (2022) |
|  |  |  | Quercetin-3-*O*-glucoside: 1.81 mg/L |  |  |
|  |  |  | Quercetin-3-*O*-rutinoside: ND |  |  |
| Barbera |  | 3.5 mg/L | Quercetin-3-*O*-glucuronide: 0.6 mg/L | 2018 | Lanati et al., (2022) |
|  |  |  | Quercetin-3-*O*-glucoside: 0.1 mg/L |  |  |
| Barbera d’Asti/ Barbera | Piemonte,Italy | 4.59 mg/L | Quercitrin: ND | 3 years | Simonetti et al., (2022) |
|  |  |  | Quercetin-3-*O*-glucoside: 1.81mg/L |  |  |
|  |  |  | Quercetin 3-*O*-rutinoside: ND |  |  |
| Barbera d’Asti/Barbera +other | Piemonte,Italy | 3.8 mg/L |  | 3 years | Buiarelli et al., (2018) |
| Barbera-Montepulciano-Sangiovese-Merlot |  | 4.59 mg/L | Rutin: 3.72 mg/mL |  | Tarola et al., (2007) |
| Barolo (1) | Italy | 1.5 mg/L | 2.6 mg/L | 1993 | McDonald et al., (-1998) |
| Basilicata IGT/ Sangiovese | Basilicata,Italy | 13.4 mg/L | Quercitrin: 0.91 mg/L | 3 years | Simonetti et al., (2022) |
|  |  |  | Quercetin-3-*O*-glucoside: 7.85 mg/L |  |  |
|  |  |  | Quercetin-3-*O*-rutinoside: ND |  |  |
| Beaujolais (2) | France | ND-1.1 mg/L* | 1.8-2.0 mg/L* | 1995 | McDonald et al., (-1998) |
| Bobal (3) | Spain | 29.08-30.28 mmol/L* | Quercetin-3-*O*-glucuronide: 20.06-28.11 mmol/L* | 2003 | Castillo-Muñoz et al., (2007) |
| Bordeaux (1) | France | 1.0 mg/L | 2.3 mg/L | 1993 | McDonald et al., (-1998) |
| Cabernet Franc |  | 0.61 mg/L |  |  | Lante et al., (2004) |
| Cabernet Franc (4) | Ontario | ≈ 2mg/L |  | 1995 | Soleas et al., (1997) |
| Cabernet Sauvignon | Bordeaux,France | 10.4 mg/L |  | 1982 | Salagoïty‐Auguste, M. H., & Bertrand, A. (1984). |
| Cabernet Sauvignon |  | 7.6 mg/L | 24.6 mg/L |  | Gardner et al., (1999) |
| Cabernet Sauvignon |  | 15.6 mg/mL |  | 1997 | Vinas et al., (2000) |
| Cabernet Sauvignon | Peloponesse, Gteece |  | Quercetin 3-*O*-rutinoside 17.3 mg/L | 1998 | Arnous et al., (2001) |
|  |  |  | Quercetin 3-*O*-rhamnoside: 16.2 mg/L |  |  |
| Cabernet Sauvignon | Suhindol, Bulgaria | 2.7 mg/L | 2.0 mg/L | 1996 | Tsanova-Savova & Ribarova (2002) |
| Cabernet Sauvignon | Targoviste, Bulgaria | 3.1 mg/L | 4.2 mg/L | 1998 | Tsanova-Savova & Ribarova (2002) |
| Cabernet Sauvignon | Svistov, Bulgaria | 3.3 mg/L | 1.4 mg/L | 1996 | Tsanova-Savova & Ribarova (2002) |
| Cabernet Sauvignon (1) | New Zeland | 1.9 mg/L | 8.1 mg/L | 1994 | McDonald et al., (1998) |
| Cabernet Sauvignon (1) | Spain | 3 mg/L | 3.3 mg/L | 1990 | McDonald et al., (1998) |
| Cabernet Sauvignon (1) | Australia | 10.2 mg/L |  | 1993 | Vuorinen et al., (2000). |
| Cabernet Sauvignon (2) | Australia | 3.0-5.5 mg/L* | 0.1-0.2 mg/L* | 1992-1994 | McDonald et al., (1998) |
| Cabernet Sauvignon (2) | California | 6.7 -10 mg/L* |  | 1994-1997 | Vuorinen et al., (2000). |
| Cabernet Sauvignon (2) | Chile | 3.0-4.4 mg/L* |  | 1997 | Vuorinen et al., (2000). |
| Cabernet Sauvignon (2) | Bulgaria | ND-1.4 mg/L* |  | 1994-1995 | Vuorinen et al., (2000). |
| Cabernet Sauvignon (2) | Chile | 25.42-28.31 mmol/L* | Quercetin-3-*O*-glucuronide: 20.85-36.85 mmol/L* | 2005-2006 | Castillo-Muñoz et al., (2007) |
|  |  |  | Quercetin-3-*O*-glucoside: ND-15.63 mmol/L* |  |  |
| Cabernet Sauvignon (3) | France | 2.6-4.6 mg/L* | 3.1-5.6 mg/L* | 1994 | McDonald et al., (1998) |
| Cabernet Sauvignon (3) | California | 1.9-7.1 mg/L* | 5.1-10.8 mg/L* | 1992-1993 | McDonald et al., (1998) |
| Cabernet Sauvignon (3) | Bulgaria | ND-1.2 mg/L* | 1.2-2.0 mg/L* | 1989-1990 | McDonald et al., (1998) |
| Cabernet Sauvignon (4) | Spain | 8.69-19.86 mmol/L* | Quercetin-3-*O*-glucuronide: 20.85-36.85 mmol/L | 2002-2005 | Castillo-Muñoz et al., (2007) |
|  |  |  | Quercetin-3-*O*-glucoside: ND-15.63 mmol/L* |  |  |
| Cabernet Sauvignon (5) | Ontario | 5.26 mg/L |  | 1995 | Soleas et al., (1997). |
| Cabernet Sauvignon (7) | California | 3.2-17.1 mg/L* |  | 1987-1991 | Frankel et al., (1995). |
| Cabernet Sauvignon (7) | Chile | 0.9-7.3 mg/L* | 3.7-21.8 mg/L* | 1992-1995 | McDonald et al., (1998) |
| Cabernet Sauvignon/Cabernet Franc | France | 8.6 mg/L |  |  | Vuorinen et al., (2000). |
| Cabernet Sauvignon/Merlot (1) | Brazil | 1.2 mg/L | 8.1 mg/L | 1994 | McDonald et al., (1998) |
| Cabernet Sauvignon/Shiraz (1) | Australia | 9.7 mg/L | 5.6 mg/L | 1994 | McDonald et al., (1998) |
| Cannonau | Sardegna,Italy | 0.40 mg/L | Quercitrin: 0.08 mg/L | 2 years | Simonetti et al., (2022) |
|  |  |  | Quercetin-3-*O*-glucoside: 2.08 mg/L |  |  |
| Carmenère (1) | Chile | 37.09 mmol/L | Quercetin-3-*O*-glucuronide: 3.72 mmol/L | 2005 | Castillo- Muñoz et al., (2007) |
|  |  |  | Quercetin-3-*O*-glucoside: 9.31 mmol/L |  |  |
| Castelli Romani DOC / Sangiovese e Montepulciano, Cesanese comune, Merlot, Nero buono | Lazio,Italy | 0.26 mg/L | Quercitrin: 0.11 mg/L | 1 years | Simonetti et al., (2022) |
|  |  |  | Quercetin-3-*O*-glucoside: 0.11 mg/L |  |  |
|  |  |  | Quercetin-3-*O*-rutinoside: ND |  |  |
| Castelli Romani/ Malvasia and Trebbiano Sangiovese e Montepulciano | Lazio,Italy | 2.03 mg/L | Quercitrin: 0.42 mg/L | 1,5 years | Simonetti et al., (2022) |
|  |  |  | Quercetin-3-*O*-glucoside: 0.17 mg/L |  |  |
| Chateauneuf du Pape (1) | France | 0.8 mg/L | 2.2 mg/L | 1993 | McDonald et al., (1998) |
| Chianti |  | 16.57 mg/L |  |  | Lante et al., (2004) |
| Chianti |  | 4.7 mg/L | 9.6 mg/L |  | Gardner et al., (1999) |
| Chianti (Sangiovese R10 Clone) | Siena, Italy | 5 mg/L | Quercetin-3-*O*-glucoside:21 mg/L |  | Ghiselli et al., (1998). |
|  |  |  | Quercetin-3-*O*-glucuronide:19 mg/L |  |  |
| Chianti Classico (2) |  | 19.57-23.54 mg/L |  |  | Lante et al., (2004) |
| Chianti Classico (4) | Italy | 7-13.2 mg/L* | 5.6-10.3 mg/L | 1991-1994 | McDonald et al., (1998) |
| Chianti/Sangiovese + other | Toscana,Italy | 4.70 mg/L | Quercitrin: 0.15 mg/L | 3 years | Simonetti et al., (2022) |
|  |  |  | Quercetin-3-*O*-glucoside: 0.06mg/L |  |  |
|  |  |  | Quercetin-3-*O*-rutinoside: ND |  |  |
| Chianti/Sangiovese+other | Toscana,Italy | 4.3 mg/L |  | 3 years | Simonetti et al., (2022) |

| Corbieres |  | 9.9 mg/L | 12.4 mg/L |  | Gardner et al., (1999) |
| --- | --- | --- | --- | --- | --- |
| Corbieres (1) | France | 5.2 mg/L | 2.6 mg/L |  | McDonald et al., (1998) |
| Cotes du Rhone |  | 2.1 mg/L | 6.4 mg/L |  | Gardner et al., (1999) |
| Cotes du Rhone (2) | France | 1.5-2.6 mg/L* | 1.9-4.3 mg/L* | 1992-1994 | McDonald et al., (1998) |
| Cotes du Roussillon Villages (1) | France | 1.2 mg/L | 3.6 mg/L | 1995 | McDonald et al., (1998) |
| Dorgan (1) | France | 0.6 mg/L | 3.5 mg/L |  | McDonald et al., (1998) |
| Dornfelder (2) | Germany | 23.67-27.34 mmol/L | Quercetin-3-*O*-glucuronide: 7.93-14.98 mmol/L* | 2004 | Castillo-Muñoz et al., (2007) |
|  |  |  | Quercetin-3-glucoside: 0.68-0.98 mmol/L* |  |  |
| Doumaine Sapt-Inour (1) | Morocco | 11.4 mg/L | 16.9 mg/L |  | McDonald et al., (1998) |
| Gaglioppo 95%-Trebbiano Toscano-Greco Bianco |  | 7.72 mg/L | Rutin: 5.80 mg/mL |  | Tarola et al., (2007) |
| Gamay Noir (3) | Ontario | 0.50 mg/L |  | 1995 | Soleas et al., (1997). |
| Gamza | Novo Selo, Bulgaria | 1.2 mg/L | 0.9 mg/L | 1995 | Tsanova-Savova & Ribarova, (2002). |
| Gamza | Lyaskovetz, Bulgaria | ND | ND | 1994 | Tsanova-Savova & Ribarova, (2002). |
| Garnacha (5) | Spain | 7.27-20.28 mmol/L* | Quercetin-3-*O*-glucuronide: 27.98-41.95 mmol/L* | 2005 | Castillo-Muñoz et al., (2007) |
| Gran Noval |  | ND |  | 1997 | Vinas et al., (2000). |
| Grenache | France | 4.5 mg/L |  | 1997 | Vuorinen et al., (2000). |
| Grenache, Mourvèdre, Syrah | France | 2.1 mg/L |  |  | Vuorinen et al., (2000). |
| Gutturnio |  | 2.33 mg/L |  |  | Lante et al., (2004). |
| Home made | Umbria,Italy | 3.32 mg/L | Quercitrin: 0.14 mg/L | 1 years | Simonetti et al., (2022) |
|  |  |  | Quercetin-3-*O*-glucoside: 0.70mg/L |  |  |
|  |  |  | Quercetin-3-*O*-rutinoside: ND |  |  |
| Jumilla |  | 12.6 mg/mL |  | 1995 | Vinas et al., (2000). |
| Kékfrankos (1) | Hungary | 1.9 mg/L | 1.7 mg/L | 1993 | McDonald et al., (1998) |
| Kotsifali | Crete,Greece | 24.90 mg/L |  |  | Sakkiadi et al., (2001). |
| Liatiko | Crete,Greece | 0.28 mg/L |  |  | Sakkiadi et al., (2001). |
| Liatiko | Crete, Greece |  | Quercetin-3-*O*-rutinoside 9.8 mg/L | 1998 | Arnous et al., (2001). |
|  |  |  | Quercetin-3-*O*-rhamnoside: 81.3 mg/L |  |  |
| Limnio | Macedonia | 3.75 mg/L |  |  | Sakkiadi et al., (2001) |
| mainly Sangiovese | Italy | 9.6 mg/L |  |  | Vuorinen et al., (2000) |
| Malbec | Bordeaux,France | 7.8 mg/L |  | 1982 | Salagoïty‐Auguste & Bertrand (1984) |
| Mavrud | Asenovgrad, Bulgaria | 4.5 mg/L | 3.1 mg/L | 1996 | Tsanova-Savova & Ribarova (2002) |
| Melnik | Damianitza, Bulgaria | 4.2 mg/L | 0.7 mg/L | 1996 | Tsanova-Savova & Ribarova (2002) |
| Melnik | Harsovo, Bulgaria | 3.2 mg/L | 1.7 mg/L | 1995 | Tsanova-Savova & Ribarova (2002) |
| Merlot | Bordeaux,France | 14.8 mg/L |  | 1982 | Salagoïty‐August & Bertrand (1984) |
| Merlot |  | 34.3 mg/mL |  | 1997 | Vinas et al., (2000). |
| Merlot | Macedonia, Greece |  | Quercetin-3-*O*-rutinoside 20 mg/L | 1998 | Arnous et al., (2001). |
|  |  |  | Quercetin-3-*O*-rhamnoside: 25.8 mg/L |  |  |
| Merlot | Stambolovo, Bulgaria | 3.2 mg/L | 4.5 mg/L | 1994 | Tsanova-Savova & Ribarova (2002) |
| Merlot | Targoviste, Bulgaria | 0.7 mg/L | 1.6 mg/L | 1998 | Tsanova-Savova & Ribarova (2002) |
| Merlot | Suhindol, Bulgaria | 2.7 mg/L | 2.0 mg/L | 1996 | Tsanova-Savova & Ribarova (2002) |
| Merlot (1) | Spain | 6.8 mg/L | 8.8 mg/L | 1993 | McDonald et al., (1998) |
| Merlot (1) | California | 6.6 mg/L |  | 1996 | Vuorinen et al., (2000) |
| Merlot (1) | Australia | 19.4 mg/L |  | 1996 | Vuorinen et al., (2000) |
| Merlot (1) | Chile | 7.1 mg/L |  |  | Vuorinen et al., (2000). |
| Merlot (1) | Bulgaria | 5.4 mg/L |  | 1995 | Vuorinen et al., (2000). |
| Merlot (2) | California | 2.1-6.8 mg/L* |  | 1990-1991 | Frankel et al., (1995) |
|  | France | 6.0-9.5 mg/L* | 5.2-7.7 mg/L* | 1994 | McDonald et al., (1998) |
| Merlot (2) | California | 2.3-4.0 mg/L* | 7.4-13.2 mg/L* | 1994-1995 | McDonald et al., (1998) |
| Merlot (2) | Spain | 8.60-12.67 mmol/L* | Quercetin-3-*O*-glucuronide: 31.44-36.55 mmol/L*  Quercetin-3-*O*-glucoside: 18.40-19.86 mmol/L* | 2005-2006 | Castillo-Muñoz et al., (2007) |
| Merlot (3) | Ontario | 2 mg/L |  | 1995 | Soleas et al., (1997) |
| Merlot (3) | Chile | 1.9-9.6 mg/L* | 6.3-14.4 mg/L* | 1993-1994 | McDonald et al., (1998) |
| Merlot (3) | Campania,Italy | 10.6-12.8 mg/L | 41.3/47.5 mg/L as sum of glycosides | 2023 | Luciano et al., (2025) |
| Merlot 89%-Montepulciano d’Abruzzo |  | 1.91 mg/L | Rutin: 3.39 mg/mL |  | Tarola et al., (2007) |
| Merlot/Pinoit Noir (1) | Bulgaria | 0.6 mg/L | 2.2 mg/L |  | McDonald et al., (1998) |
| Minervois |  | 4.8 mg/L | 7.9 mg/L |  | Gardner et al., (1999) |
| Minervois (2) | France | 5.8-15.8 mg/L* | 5.0-13.1 mg/L* | 1994 | McDonald et al., (1998) |
| Montepulciano-Cesanese |  | 1.53 mg/L | Rutin: 5.77 mg/mL |  | Tarola et al., (2007) |
| Morellino di Scansano/ 85% Sangiovese+15% other | Toscana,Italy | 0.16 mg/L | Quercitrin: ND | 3 years | Simonetti et al., (2022) |
|  |  |  | Quercetin-3-*O*-glucoside: ND |  |  |
|  |  |  | Quercetin 3-*O*-rutinoside: ND |  |  |
| Moschomavro | Peloponnese,Greece | 10.29 mg/L |  |  | Sakkiadi et al., (2001) |
| Negro Amaro-Malvasia |  | 3.92 mg/L | Rutin: 2.63 mg/mL |  | Tarola et al., (2007) |
| Nero d'Avola | Calabria, Italy | 16.21 mg/L | Quercitrin: 0.02 mg/L | 5 years | Simonetti et al., (2022) |
|  |  |  | Quercetin-3-*O*-glucoside: 1.41 mg/L |  |  |
|  |  |  | Quercetin-3-*O* rutinoside: ND |  |  |
| Nero d'Avola (2) | Sicilia,Italy | ND-0.5 mg/L* |  | 6 years | Buiarelli et al., (2018) |
| Nero d'Avola | Puglia,Italy | 12.4 mg/L | 135.16 mg/L | 2023 | Luciano et al., (2025) |

| Pallagrello Nero (6) | Italy | 5.6-8.1 mg/L* | 11.8-12.4 mg/L* | 2012 | Gambuti et al.,2020 |
| --- | --- | --- | --- | --- | --- |
| Pallagrello Nero | Campania,Italy | 9.4 mg/L | 51.3 mg/L as sum of glycosides | 2023 | Luciano et al., (2025) |
| Petit Shiraz | California | 7.7 mg/L |  | 1992 | Teissedre et al., (1996). |
| Petit Shiraz (3) | California | 3.2-13.2 mg/L* |  | 1987-1990 | Frankel et al., (1995). |
| Petit Verdot (1) | France | 31.09 mmol/L | Quercetin-3-*O*-glucuronide: 11.71 mmol/L | 2004 | Castillo-Muñoz et al., (2007) |
|  |  |  | Quercetin-3-*O*-glucoside: 4.04 mmol/L |  |  |
| Petit Verdot (2) | Spain | 2.59-25.56 mmol/L* | Quercetin-3-*O*-glucuronide: 22.26-24.99 mmol/L* | 2004-2005 | Castillo-Muñoz et al., (2007) |
|  |  |  | Quercetin-3-*O*-glucoside: 1.15-2.08 mmol/L* |  |  |
| Pinot Noir (1) | California | 12.9 mg/L |  | 1992 | Frankel et al., (1995). |
| Pinot Noir (1) | Oregon | 1.9 mg/L | 3.0 mg/L | 1994 | McDonald et al., (1998) |
| Pinot Noir (1) | Romania | 3 mg/L | 2.9 mg/L | 1990 | McDonald et al., (1998) |
| Pinot Noir (2) | Chile | 5.9-6.5 mg/L* | 10.6-13.2 mg/L* | 1995 | McDonald et al., (1998) |
| Pinot Noir (2) | California | 2.8-3.8 mg/L* | 4.5-5.3 mg/L* | 1993 | McDonald et al., (1998) |
| Pinot Noir (3) | France | 0.9-2.6 mg/L* | 2.6-3.8 mg/L* | 1992-1994 | McDonald et al., (1998) |
| Pinot Noir (6) | Ontario | 2.60 mg/L |  | 1995 | Soleas et al., (1997). |
| Pinot Noit |  | 1.5 mg/L | 1.5 mg/L |  | Gardner et al., (1999) |
| Primitivo |  | 1.18 mg/mL | Rutin: 7.78 mg/mL |  | Tarola et al., (2007) |
| Primitivo | Puglia,Italy | 5.39 mg/L | Quercitrin: 0.12 mg/L | 1 years | Simonetti et al., (2022) |
|  |  |  | Quercetin-3-*O*-glucoside: 0.65 mg/L |  |  |
|  |  |  | Quercetin 3-*O*-rutinoside: ND |  |  |
| Primitivo | Puglia,Italy | 5.3 mg/L | 68.5 mg/L as sum of glycosides | 2023 | Luciano et al., (2025) |
| Primitivo di Manduria |  | ND | Rutin: 2.76 mg/mL |  | Tarola et al., (2007) |
| Puglia IGT/ Lambrusco Maestri | Puglia,Italy | 0.66 mg/L | Quercitrin: 0.06 mg/L | 1 years | Simonetti et al., (2022) |
|  |  |  | Quercetin-3-glucoside: 0.17 mg/L |  |  |
|  |  |  | Quercetin 3-*O-*rutinoside: ND |  |  |
| Red Bordeaux mix | Bordeaux,France | 4.1 mg/L |  | 1990 | Hertog et al., (1993). |
| Red California/Dry Pinot Noir | California | 8.8 mg/L |  | 1990 | Hertog et al., (1993). |
| Red Chianti | Italy | 16 mg/L |  | 1990 | Hertog et al., (1993). |
| Red Cirò (Gaglioppo) |  | 3.0 mg/L | Quercetin-3-*O*-glucuronide: 2.4 mg/L | 2014 | Lanati et al., (2022) |
|  |  |  | Quercetin-3-*O*-glucoside: 0.7 mg/L |  |  |
| Red Rioja | Spain | 4.1mg/L |  | 1990 | Hertog et al., (1993). |
| Rioja (1) | Spain | ND | 2.2 mg/L |  | McDonald et al., (1998) |
| Rodrejo |  | 22.7 mg/mL |  | 1997 | Vinas et al., (2000). |
| Sabatacha |  | ND |  | 1995-1997 | Vinas et al., (2000). |
| Salice Salentino / 75% Negroamaro +other | Puglia,Italy | 1.10 mg/L | Quercitrin: 0.17 mg/L | 2 years | Simonetti et al., (2022) |
|  |  |  | Quercetin-3-glucoside: 0.01 mg/L |  |  |
|  |  |  | Quercetin 3-*O* rutinoside: ND |  |  |
| Salice Talentino/ (Negroamaro+other mix) | Puglia,Italy | 1.5 mg/L |  | 2 years | Buiarelli et al., (2018) |
| Sangiovese | Emilia-Romagna,Italy | 6.05 mg/L | Quercitrin: 0.10 mg/L | 3 years | Simonetti et al., (2022) |
|  |  |  | Quercetin-3-glucoside: 0.51 mg/L |  |  |
|  |  |  | Quercetin 3-*O-*rutinoside: ND |  |  |
| Sangiovese |  | 11.3 mg/L | Quercetin-3-glucuronide: 6 mg/L | 2014 | Lanati et al., (2022) |
|  |  |  | Quercetin-3-glucoside: 2.9 mg/L |  |  |
| Sangiovese (1) | Italy | 36.05 mmol/L | Quercetin-3-glucuronide: 13.23 mmol/L | 2004 | Castillo-Muñoz et al., (2007) |
|  |  |  | Quercetin-3-glucoside: 1.41 mmol/L |  |  |
| Sangiovese (1) | Lazio,Italy | 0.5 mg/L |  | 3 years | Buiarelli et al., (2018) |
| Sangiovese (1) | Basilicata,Italy | 0.7 mg/L |  | 3 years | Buiarelli et al., (2018) |
| Sangiovese (1) | Marche, Italy | 0.3 mg/L |  | 3 years | Buiarelli et al., (2018) |
| Sangiovese (22) | Italy | 0.4-8.6 mg/L* | 3.1-33.9 mg/L* | 2015-2016-2017-2018 | Gambuti et al., (2020) |
| Sangiovese (3) | Italy | 16.0-17 mg/L | 56-101.8 mg/L as sum of glycosides | 2023 | Luciano et al., (2025) |
| Sangiovese 60%-Cabernet-Ciliegiolo |  | 1.70 mg/L | Rutin: 3.22 mg/mL |  | Tarola et al., (2007) |
| Shiraz-Merlot-Cabernet Sauvignon-Petit Verdot |  | 5.40 mg/mL | Rutin: 5.51 mg/mL |  | Tarola et al., (2007) |
| Solopaca |  | 4.28 mg/L | Rutin: 6.67 mg/mL |  | Tarola et al., (2007) |
| Spanish wine | Spain | 12 mg/L | Quercitrin: ND |  | Simonetti et al., (2022) |
| Spanish wine | Spain | 13 mg/L | Quercetin-3-glucoside: 2.86 mg/L |  | Simonetti et al., (2022) |
| Spanish wine | Spain | 14 mg/L | Quercetin 3-*O*-rutinoside: 1.67 mg/L |  | Simonetti et al., (2022) |
| Syrah |  | 43.1 mg/mL |  | 1997 | Vinas et al., (2000). |
| Syrah | Macedonia,Greece | 12.07 mg/L |  |  | Sakkiadi et al., (2001). |
| Syrah | Macedonia, Greece |  | Quercetin 3-*O*-rutinoside 31 mg/L | 1998 | Arnous et al., (2001) |
|  |  |  | Quercetin 3-*O*-rhamnoside: 122.6 mg/L |  |  |
| Syrah | Trentino,Italy | 0.40 mg/L | Quercitrin: 0.91 mg/L | 6 months | Simonetti et al., (2022) |
|  |  |  | Quercetin-3-glucoside: 7.85 mg/L |  |  |
|  |  |  | Quercetin 3-*O*-rutinoside: ND |  |  |
| Syrah (2) | Spain | 11.92-17.89 mmol/L* | Quercetin-3-glucuronide: 17.59-25.03 mmol/L* | 2003-2004 | Castillo-Muñoz et al., (2007) |
|  |  |  | Quercetin-3-glucoside: 0.68-9.65 mmol/L* |  |  |
| Tempranillo |  | 30 μg/mL |  | 1997 | Vinas et al., (2000) |
| Tempranillo (1) | Spain | 0.1 mg/L | 3 mg/L | 1994 | McDonald et al., (1998) |
| Tempranillo (7) | Spain | 1.69-17.80 mmol/L* | Quercetin-3-glucuronide: 13.56-20.15 mmol/L* | 1990-2006 | Castillo-Muñoz et al., (2007) |
|  |  |  | Quercetin-3-glucoside: ND-16.17 mmol/L* | 1990-2007 |  |
| Valpolicella |  | 0.5 mg/L | 3.6 mg/L |  | Gardner et al., (1999) |
| Valpolicella |  | 0.53 mg/L |  |  | Lante et al., (2004) |
| Valpolicella (2) | Italy | ND-0.3 mg/L* | 4.6-5-5 mg/L* | 1994 | McDonald et al., (1998) |
| Vertzami | Lefkada | 0.85 mg/L |  |  | Sakkiadi et al., (2001) |
| Xinomavro | Macedonia, Greece |  | Quercetin 3-O rutinoside 8.0-13.2 mg/L* | 1998 | Arnous et al., (2001) |
|  |  |  | Quercetin 3-O-rhamnoside: 12.2-25.4 mg/L* |  |  |

Number of analysed wines in parentheses. ^§^The table contains all the information available from the consulted papers.

When present, * indicate the minimum and maximum values above all the samples into the brackets.

**^#^** When glycosides are not specified, it is because they were not indicated in the reference article.

**Supplementary References for Table S1:**

Arnous, A., Makris, D. P., & Kefalas, P. (2001). Effect of principal polyphenolic components in relation to antioxidant characteristics of aged red wines. *Journal of agricultural and food chemistry*, 49(12), 5736-5742.

Buiarelli, F., Bernardini, F., Di Filippo, P., Riccardi, C., Pomata, D., Simonetti, G., & Risoluti, R. (2018). Extraction, purification, and determination by HPLC of Quercetin in some Italian wines. *Food Analytical Methods*, *11*(12), 3558-3562.

Castillo-Muñoz, N., Gómez-Alonso, S., García-Romero, E., & Hermosín-Gutiérrez, I. (2007). Flavonol profiles of *Vitis vinifera* red grapes and their single-cultivar wines. *Journal of agricultural and food chemistry*, *55*(3), 992-1002.

Frankel, E. N., Waterhouse, A. L., & Teissedre, P. L. (1995). Principal phenolic phytochemicals in selected California wines and their antioxidant activity in inhibiting oxidation of human low-density lipoproteins. *Journal of Agricultural and Food chemistry*, *43*(4), 890-894.

Gardner, P. T., McPhail, D. B., Crozier, A., & Duthie, G. G. (1999). Electron spin resonance (ESR) spectroscopic assessment of the contribution of quercetin and other flavonols to the antioxidant capacity of red wines. *Journal of the Science of Food and Agriculture*, *79*(7), 1011-1014.

Ghiselli, A., Nardini, M., Baldi, A., & Scaccini, C. (1998). Antioxidant activity of different phenolic fractions separated from an Italian red wine. *Journal of agricultural and food chemistry*, *46*(2), 361-367.

Hertog, M. G., Hollman, P. C., & Van de Putte, B. (1993). Content of potentially anticarcinogenic flavonoids of tea infusions, wines, and fruit juices. *Journal of agricultural and food chemistry*, 41(8), 1242-1246.

Lanati, D., Cascio, P., Pollon, M., Corona, O., & Marchi, D. (2022). Solubility of quercetin in wines. *South African Journal of Enology and Viticulture*, *43*(2), 146-156.

Lante, A., Crapisi, A., Lomolino, G., & Spettoli, P. (2004). Chemical parameters, biologically active polyphenols and sensory characteristics of some Italian organic wines. *Journal of Wine Research*, *15*(3), 203-209.

Luciano, A., Gambuti, A., Moio, L., & Picariello, L. (2025). Screening of Italian red wines for quercetin precipitation risk index: This is an original research article submitted in cooperation with Macrowine 2025. *OENO One*, *59*(2).

McDonald, M. S., Hughes, M., Burns, J., Lean, M. E., Matthews, D., & Crozier, A. (1998). Survey of the free and conjugated myricetin and quercetin content of red wines of different geographical origins. *Journal of agricultural and food chemistry*, 46(2), 368-375.

Pavia, C., Bufo, S. A., Scopa, A., Scrano, L., Guerrieri, A., & Cataldi, T. R. (2001). Determination of phenolic compounds of biological interest in some Italian red wines by HPLC-DAD. *Advances in food sciences*, *23*(3), 100-107.

Sakkiadi, A. V., Stavrakakis, M. N., & Haroutounian, S. A. (2001). Direct HPLC assay of five biologically interesting phenolic antioxidants in varietal Greek red wines. *LWT-Food Science and Technology*, *34*(6), 410-413.

Salagoïty‐Auguste, M. H., & Bertrand, A. (1984). Wine phenolics—analysis of low molecular weight components by high performance liquid chromatography. *Journal of the Science of Food and Agriculture*, *35*(11), 1241-1247.

Simonetti, G., Buiarelli, F., Bernardini, F., Di Filippo, P., Riccardi, C., & Pomata, D. (2022). Profile of free and conjugated quercetin content in different Italian wines. *Food Chemistry*, 382, 132377.

Soleas, G. J., Dam, J., Carey, M., & Goldberg, D. M. (1997). Toward the fingerprinting of wines: cultivar-related patterns of polyphenolic constituents in Ontario wines. *Journal of Agricultural and Food Chemistry*, *45*(10), 3871-3880.

Tarola, A. M., Milano, F., & Giannetti, V. (2007). Simultaneous determination of phenolic compounds in red wines by HPLC‐UV. Analytical Letters, 40(12), 2433-2445.

Teissedre, P. L., Frankel, E. N., Waterhouse, A. L., Peleg, H., & German, J. B. (1996). Inhibition ofIn vitrohuman LDL oxidation by phenolic antioxidants from grapes and wines. *Journal of the Science of Food and Agriculture*, *70*(1), 55-61.

Tsanova-Savova, S., & Ribarova, F. (2002). Free and conjugated myricetin, quercetin, and kaempferol in Bulgarian red wines. *Journal of Food Composition and Analysis*, *15*(6), 639-645.

Vinas, P., Lopez-Erroz, C., Marın-Hernandez, J. J., & Hernandez-Cordoba, M. (2000). Determination of phenols in wines by liquid chromatography with photodiode array and fluorescence detection. *Journal of Chromatography A*, *871*(1-2), 85-93.

Vuorinen, H., Määttä, K., & Törrönen, R. (2000). Content of the flavonols myricetin, quercetin, and kaempferol in Finnish berry wines. *Journal of Agricultural and Food Chemistry*, *48*(7), 2675-2680.
